# Rotation of the *c*‐Ring Promotes the Curvature Sorting of Monomeric ATP Synthases

**DOI:** 10.1002/advs.202301606

**Published:** 2023-09-13

**Authors:** David Valdivieso González, Marcin Makowski, M. Pilar Lillo, Francisco J. Cao‐García, Manuel N. Melo, Víctor G. Almendro‐Vedia, Iván López‐Montero

**Affiliations:** ^1^ Departamento Química Física Universidad Complutense de Madrid Avda. Complutense s/n Madrid 28040 Spain; ^2^ Instituto de Investigación Biomédica Hospital Doce de Octubre (imas12) Avenida de Córdoba s/n Madrid 28041 Spain; ^3^ Instituto de Medicina Molecular Facultade de Medicina Universidade de Lisboa Lisbon 1649‐028 Portugal; ^4^ Instituto de Tecnologia Química e Biológica António Xavier Universidade Nova de Lisboa Av. da República Oeiras 2780‐157 Portugal; ^5^ Departamento Química Física Biológica Instituto de Química‐Física “Blas Cabrera” (CSIC) Serrano 119 Madrid 28006 Spain; ^6^ Departamento de Estructura de la Materia Física Térmica y Electrónica Universidad Complutense de Madrid Plaza de Ciencias 1 Madrid 28040 Spain; ^7^ Instituto Madrileño de Estudios Avanzados en Nanociencia IMDEA Nanociencia C/ Faraday 9 Madrid 28049 Spain; ^8^ Instituto Pluridisciplinar Paseo Juan XXIII 1 Madrid 28040 Spain

**Keywords:** *E. coli*, F_1_F_o_ ATP synthase, giant vesicles, lipid nanotubes, micromanipulation

## Abstract

ATP synthases are proteins that catalyse the formation of ATP through the rotatory movement of their membrane‐spanning subunit. In mitochondria, ATP synthases are found to arrange as dimers at the high‐curved edges of *cristae*. Here, a direct link is explored between the rotatory movement of ATP synthases and their preference for curved membranes. An active curvature sorting of ATP synthases in lipid nanotubes pulled from giant vesicles is found. Coarse‐grained simulations confirm the curvature‐seeking behaviour of rotating ATP synthases, promoting reversible and frequent protein‐protein contacts. The formation of transient protein dimers relies on the membrane‐mediated attractive interaction of the order of 1.5 *k_B_T* produced by a hydrophobic mismatch upon protein rotation. Transient dimers are sustained by a conic‐like arrangement characterized by a wedge angle of θ ≈ 50°, producing a dynamic coupling between protein shape and membrane curvature. The results suggest a new role of the rotational movement of ATP synthases for their dynamic self‐assembly in biological membranes.

## Graphical abstract

An active curvature sorting of monomeric ATP synthases into lipid nanotubes is measured under rotating conditions. As suggested by coarse‐grained simulations, the rotational movement of proteins produces a hydrophobic mismatch at the protein–lipid interface leading to a membrane‐mediated attractive interaction among proteins. As a result, transient dimers with a conic‐like arrangement are formed, producing a dynamic coupling between protein shape and membrane curvature.

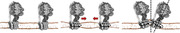

## 1 Introduction

**Figure 1:**
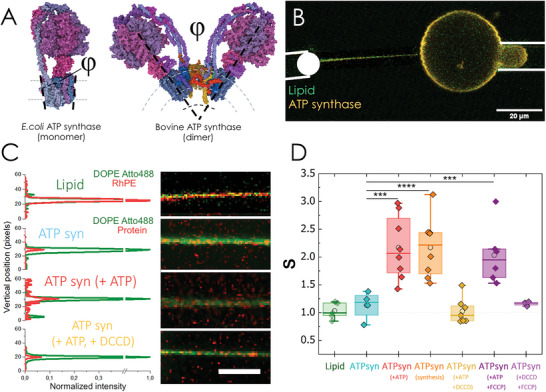
A) Structure of ATP synthase from *Escherichia coli* (left) and *Bos taurus* (right). The bacterial protein is monomeric, whereas the bovine counterpart forms dimers sustained by the additional subunits *e* (yellow), *f* (orange), *g* (red) and *j* (brown). φ is the wedge angle between monmomers that might induce the local bending of the membrane (dashed lines). B) Confocal micrograph of a tube pulled from a GUV containing reconstituted ATP synthases. The membrane (green channel) was labelled with the fluorescent lipid DOPE Atto‐488, whereas the protein was labelled with Alexa 555. A first micropipette holds a biotinylated silica beads and pulls a membrane tube from a GUV, which is seized by a second micropipette. The relative concentrations of lipid and protein in the tube and in the GUV are measured by confocal fluorescence microscopy (see Methods). C) Confocal images of different tubes pulled from pure lipid GUVs (row 1) and proteoGUVs containing ATP synthase in passive conditions (row 2), upon incubation with ATP (row 3) or in the presence of both ATP and the rotation inhibitor DCCD (row 4). Fluorescence intensiy of lipid and protein channels are normalized to the lipid value. Scale bar is 5 µm. D) Box plots comparing the sorting ratio for tubes pulled from GUVs ( *r_t_* =  50 ± 20 nm) containing the red fluorescent lipid Rhodamine PE (Lipid), ATP synthase (ATPsyn), ATPsyn with ATP, ATPsyn in the synthesis mode, ATPsyn with ATP and DCCD, ATPsyn with ATP and FCCP and ATPsyn with DCCD and FCCP. The median is represented with a line; the box represents the 25th to 75th percentiles; and error bars show the 5th–95th percentile. The average sorting ratios (cercles) for the lipid control, ATPsyn, ATPsyn with ATP and DCCD and ATPsyn with DCCD and FCCP are similar (S=1.1), whereas ATPsyn in rotating conditions is enriched in the tubes (S>2). Statistical significance: *** (*p* ≤ 0.001); **** (*p* ≤ 0.0001).

F_1_F_o_‐ATP synthase (ATP synthase) is an essential transmembrane protein that synthesizes the biochemical energy of the cell, i.e., adenosine triphosphate (ATP).^[^ ^1^ ^]^ Structurally highly conserved among different organisms, the ATP synthase protein is composed of two multi‐subunit complexes: the soluble catalytic head F_1_, and the membrane domain F_o_.^[^ ^2^ ^]^ The F_o_ domain contains an homo‐oligomeric *c*‐ring, which displays a rotatory movement generally triggered by a proton (ΔpH) or a electrochemical (ΔΨ) gradient across the membrane and transmitted to the catalytic part of F_1_ through a central stalk.^[^ ^3^ ^]^ The spinning movement of the central stalk produces conformational changes in the F_1_ head leading to the synthesis of ATP from adenosin diphosphate (ADP) and inorganic phosphate (P_i_).^[^ ^4^ ^]^ Remarkably, an excess in the ratio of ATP/ADP produces a reverse movement of the *c*‐ring and protons are shuttled in the opposite direction. In addition to the central stalk, the catalytic head of F_1_ is linked to the F_o_ domain through a peripheral stalk, thus preventing the rotation of the whole assembly. The rotor rings of ATP synthases have circular symmetry and contain 8 to 15 subunits depending on the organism.^[^ ^5^ ^]^ However, the major structural difference between eukaryotic and prokaryotic ATP synthases is the presence of additional subunits found at the vicinity of the peripheral stalk. Some of these subunits are involved in the ATP synthase dimer formation.^[^ ^6^ ^]^ Thus, the mitochondrial ATP synthase form dimers in the membrane,^[^ ^7^, ^8^ ^]^ whereas the prokaryotic counterpart is thought to occur exclusively as a monomer (**Figure** **1**A).

A remarkable consequence of ATP synthase dimerization is the ability of protein dimers to accumulate into highly curved membranes,^[^ ^9^ ^]^ which is necessary for the promotion and maintenance of the high‐curvature edges of the mitochondrial inner membrane; i.e., *cristae*.^[^ ^10^ ^]^ The conic‐like arrangement of dimers is characterized by a wedge angle that varies from φ ≈ 55° to 120°, which might induce the local bending of the membrane,^[^ ^7^, ^8^ ^]^ (Figure 1A). Moreover, the deletion of the specific subunits leading to mitochondrial ATP synthase dimerization alters the *cristae* structure to a balloon‐like shape.^[^ ^8^ ^]^ Interestingly, the cross‐linking of ATP synthase dimers prevents the formation of mitochondrial *cristae* in vivo,^[^ ^11^ ^]^ suggesting that additional factors to protein dimerization might be necessary for curvature generation and *cristae* formation. Recently, we have proposed the ATP synthase activity as a complementary mechanism for membrane remodelling based on the rotatory movement of the protein.^[^ ^12^ ^]^ However, the experimental demonstration for an active protein sorting into curved membranes is still lacking. In the present work we provide evidence for a rotary‐mediated curvature sorting of monomeric ATP synthases. By combining vesicle pulling experiments with molecular dynamics simulations, we gathered data that enabled the determination of the intermolecular interactions involved in this process.

## 2 Results

### 2.1 ATP Synthase Sorting in Giant Vesicle Tethers

We first investigated the protein sorting of fluorescently labelled ATP synthases into lipid nanotubes pulled from giant unilamellar vesicles (GUVs) made of 1‐Palmitoyl‐2‐oleoyl‐sn‐glycero‐3‐phosphocholine (POPC). Tether pulling experiments are widely used for studying the curvature sorting and curvature sensing of lipids and proteins,^[^ ^13^, ^14^ ^]^ as a function of the lipid composition, protein density or tube diameter. In a typical experiment for curvature sorting of membrane proteins, a biotinylated silica bead and a GUV, containing the fluorescent membrane protein, a membrane lipid dye and biotinylated lipids, are held by two micropipettes under a confocal microscope (Figure 1B). Then the bead is brought into contact with the proteo‐GUV membrane and streptavidin‐biotin bonds form, leading to a strong adhesion between the membrane and the bead. Upon separation of the bead, a tube is pulled from the membrane as previously described.^[^ ^15^ ^]^ The relative density of the protein in the tube and in the proteo‐GUV, i.e., the protein sorting *S*, can be obtained through the equation^[^ ^13^ ^]^:

(1) $$S\;=\frac{PCF_{1}\times \frac{I_{tube}^{prot}}{I_{GUV}^{prot}}}{PCF_{2}\times \frac{I_{tube}^{lip}}{I_{GUV}^{lip}}}\;=\;\frac{\phi_{tube}}{\phi_{GUV}}$$

where $I_{tube}^{prot}$ and $I_{GUV}^{prot}$ are the fluorescence intensities of the fluorescent protein in the tube and the GUV respectively and $I_{tube}^{lip}$ and $I_{GUV}^{lip}$ are the fluorescence intensities of the lipid dye in the tube and the GUV respectively (Figure 1C). PCF_*i*_ is a correction factor for the effects of the light polarization on probe *i*. To demonstrate the active contribution of ATP synthase as a spontaneous curvature generator we used monomeric ATP synthases purified from *Escherichia coli* (*E.coli*). This allowed us to discard any biochemical specific interaction between ATP synthase subunits leading to protein dimerization (i.e., spontaneous curvature generation) and subsequent partition into curved membranes in the absence of protein rotation.

We obtained lipid tubes ranging from 13 to 135 nm in radius after pulling out and in several times the biofunctionalized bead previously brought into contact with the GUV (see Methods). For tubes with radius, $r_{t}=50\pm 20\;\mathrm{nm}$; the intensity profile ratio of the protein channel (orange) and the lipid channel (green) were similar in the absence of ATP, $S_{ATPsyn}=1.1\pm 0.2\;\left(N=4\right)$ (Figure 1D). The monomeric ATP synthase was not sorted into highly curved bilayers or in other words, the membrane composition of the tube and the GUV is the same. When incubated with ATP, i.e., under rotating conditions, the fluorescence ratio of the protein increases, whereas the normalized intensity of the lipid remains unaltered, yielding a sorting ratio of $S_{ATPsyn}^{hydrolysis}=2.2\pm 0.6\;\left(N=8\right)$ (Figure 1D). Although modest (2‐fold), the sorting value significantly larger than unity proved the enrichment of ATP synthases in the lipid tubes. Interestingly, a sorting ratio of $S_{ATPsyn}^{synthesis}=2.2\pm 0.5\left(N=8\right)$ was also obtained in the synthesis mode, where ATP synthase was activated upon incubation with the selective K^+^ ionophore valinomycin in the presence of a KCl concentration gradient across the membrane of proteoGUVs (higher outside).^[^ ^16^ ^]^ To allocate the action of protein rotation to the observed increased sorting, we performed a control experiment incubating simultaneously with ATP and N,N′‐dicyclohexylcarbodiimide (DCCD, 1 mM final concentration). DCCD is a classical inhibitor of the ATP synthase, which binds covalently to the *c*‐ring in F_o_ and thereby blocks its rotation.^[^ ^17^ ^]^ At this high concentration, DCCD might also inhibit F_1_^[^ ^18^, ^19^ ^]^ through its binding to the βGlu192. However, the inhibitory effect on F_1_ by DCCD requires a Mg^2+^‐free buffer,^[^ ^20^ ^]^ but this cation is present in the sorting buffers (see Figure S1, Supporting Information, for details). Under exclusive F_o_ inhibitory conditions, the sorting parameter was similar to that measured in the absence of ATP, $S_{ATPsyn}^{hydrolysis+DCCD}=1.0\pm 0.2\;\left(N=8\right)$ (Figure 1D).

The emergence of a curvature sorting might be supported by the proton pump activity of ATP synthase through the membrane‐spanning component. A localized pH gradient would be the driving force for a preferred spontaneous curvature across the membrane^[^ ^21^ ^]^ through a change in the lipid packing on one side of the membrane.^[^ ^22^ ^]^ The driving force for the seeking curvature behaviour of ATP synthases might be also maintained by a diffusive flux of protons along the membrane^[^ ^23^ ^]^ as suggested previously.^[^ ^24^ ^]^ To assess the proton gradient across and along the membrane as an important mechanism for the observed protein sorting, additional experiments were performed where the filaments were pulled under the action of the widely used protonophore FCCP. We first considered the scenario where the ATP synthase would still rotate in the presence of ATP, but without establishing a proton gradient across the membrane due to the dissipative action of FCCP. Remarkably, the direct effect of rotation on the ATP synthase sorting in lipid nanotubes was confirmed by a sorting ratio of $S_{ATPsyn}^{hydrolysis+FCCP}=2.0\pm 0.5\;\left(N=6\right)$. Then, we also evaluated the curvature sorting of ATP synthase in the presence of a pH gradient across the lipid bilayer (basic inside) promoted by the incubation of FCCP under non‐active conditions. Due to the dissimilar volume enclosed by the tube as compared to the volume of the GUV, proton translocation across the membrane through FCCP might create an additional proton gradient along the plane of the membrane (acid in the tube). To prevent the rotation of the proteins and uncouple both contributions, the system was also incubated with DCCD and the rotation of the *c*‐ring was inhibited accordingly. Again, the sorting parameter was close to one under these conditions, $S_{ATPsyn}^{FCCP+DCCD}=1.2\pm 0.1\;\left(N=4\right)$ (Figure 1D), so our results suggest there is no direct correlation between protein sorting and a proton gradient across and along the membrane.

An additional evaluation of the direct effect of proton pumping on the protein sorting in lipid nanotubes (uncoupled with rotatory movement) was experimentally addressed using GUVs containing Bacteriorhodopsin (BR) from *Halobacterium salinarum*. BR is a light‐driven proton pump that produces a trans‐membrane stable electrochemical gradient of protons. The pH gradient‐dependent sorting of BR was evaluated after illuminating the sample in the presence of valinomycin and a trans‐membrane [K^+^] gradient (higher outside) to overcome the retroinhibitory effect of the electrochemical gradient by the proton pumping activity of BR.^[^ ^25^ ^]^ Under those conditions, the sorting parameter of BR was $S_{BR}^{light}=1.1\pm 0.2\;\left(N=6\right)$, which is similar to the sorting parameter obtained without illuminating the sample, $S_{BR}^{dark}=1.1\pm 0.2\;\left(N=7\right)$. This experiment confirms that a proton gradient across the membrane does not promote the protein sorting into curved filaments (see Figure S2, Supporting Information, for details).

Finally, we measured also an even distribution of the fluorescent lipid RhPE with $S_{l}=1.0\pm 0.1\;\left(N=5\right)$ (Figure 1D). Lipids prone to form planar bilayers, i.e., with vanishing spontaneous curvature, or far from a demixing point are not affected by membrane curvature,^[^ ^13^, ^26^ ^]^ Overall, we measured an enrichment of ATP synthase in curved membranes only under rotating conditions. To our knowledge, we detected  for the first time an active protein sorting in nanometric lipid tubes.

### 2.2 Molecular Modelling of Rotation‐Induced ATP Synthase Sorting

**Figure 2:**
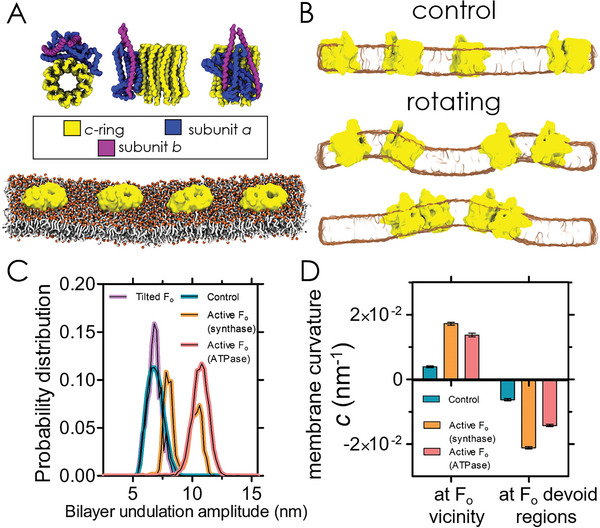
MD simulations reveal the generation of out of equilibrium bilayer fluctuations and curvature preference of rotating transmembrane F_o_ domains. A) Top: overview of the structure of the F_o_ transmembrane domain of the ATP synthase and its subunits; Bottom: Snapshots of the simulated system at its initial configuration. The choline headgroups of the POPC are coloured in orange, the acyl chains are coloured in light grey, and F_o_ transmembrane domains in yellow. Solvent molecules have been removed from the visualization for clarity. B) Representative snapshots of the bilayer with passive (control) and active (rotating) F_o_ domains. The collective rotation induces bilayer fluctuations, which are negligible in the control. C) Probability distribution of the spreads between the lipid phosphate *z*‐coordinate maxima and minima, indicating that the rotating protein has a stronger tendency to generate bilayer fluctuations. D) Local curvature of the bilayer regions in the vicinity of the F_o_ domain versus the regions devoid of protein, indicating a strong tendency of the protein to position in regions with positive curvature. The bars represent a 95% confidence interval, obtained from bootstrap resampling with subsampling to account for time‐series autocorrelation.

To get deeper insight into the mechanism behind the active curvature sorting of ATP synthases in lipid tubes, we ran coarse‐grained (CG) molecular dynamics simulations employing the state‐of‐the‐art Martini 3 model.^[^ ^27^ ^]^ For simplicity, and because we focused on events at the membrane level, only the *c*‐ring and the transmembrane segment of the F_o_ peripheral stalk (*a* and *b* subunits) were simulated (**Figure** **2**A). As in case of experiments, the structure of the *E. coli* ATP synthase was used and proteins were embedded in a POPC membrane. To keep a similar lipid‐to‐protein ratio used in experiments $\left(L/P\approx 5\;\times \;10^{3}\right)$ ^[^ ^16^ ^]^ we simulated a system composed of 4 proteins in a ≈ 52  ×  13 nm^2^ sized lipid bilayer, containing ≈ 2000 POPC lipids (Figure 2A). A torque was then applied on the *c*‐ring of the rotors relative to their peripheral stalk, to mimic the rotation caused by ATP hydrolysis. We employed a torque of enough magnitude that rotation could be observed in the microsecond timescale accessible to the CG simulations.^[^ ^28^ ^]^ This resulted in average rotational velocities of ω_*CG*_ ≈ 470 kHz, which is three orders of magnitude times higher than the time‐averaged rotational rates measured for 40‐nm beads attached to the F_1_‐domain through the γ subunit (ω_*exp*_ ≈ 200 Hz).^[^ ^29^ ^]^ While such a difference in rotational velocity may impact the representativity of the CG simulations, we note that rotation still proceeds step‐wise, with room to thermally wiggle, often with several nanosecond intervals between steps (see Figure S3, Supporting Information, and compare with the behaviour at lower and higher torques, where either no rotation is seen, or is seen in a different dynamic regime). This indicates that the progression still displays thermal dependence, as is expected for the real system. Additionally, we also ran simulations in the ATP synthesis mode by applying an opposite torque to mimic the rotation caused by proton translocation. Control simulations were run in the absence of applied torque.

Strikingly, rotation of *c*‐rings quickly promoted membrane buckling with high amplitudes (Figure 2B; Movies S1 and S2, Supporting Information). Non‐equilibrium membrane fluctuations have been observed experimentally in proteo‐GUVs containing the ATP synthase in active conditions.^[^ ^16^ ^]^ By contrast, control simulations where no torque was applied displayed only transient, low‐amplitude thermal undulations (Figure 2B; Movie S3, Supporting Information); thermal undulations, which occur in simulated and real membranes alike, are the result of stochastic, off‐plane lipid movement occurring preferentially along the membrane's bending modes.^[^ ^30^ ^]^ The statistical characterization of the membrane fluctuations was performed by building the probability distribution of the maximum peak‐to‐peak undulation amplitude in the *z*‐direction (Figure 2C), calculated from the coordinates of the POPC phosphates throughout the trajectory and over the 4 independent runs for each condition (see Supporting Information for details). In the absence of rotation and as expected for thermal undulations (Figure 2B, top), membrane fluctuations displayed a distribution with mean value 〈*h*〉  =  6.9 nm and standard deviation *SD*  =  0.65 nm. In contrast, when applying a torque in the hydrolysis mode, the peak‐to‐peak distribution shifted to higher amplitudes (11 nm,  *SD*  =  0.6 nm) than the control case. Interestingly, the torque applied in the synthesis mode splits the peak‐to‐peak distribution into 2 maxima (at ≈ 8 nm and 11 nm). In both synthesis and hydrolysis operating modes, the shifted distributions are consistent with the larger fluctuations explored by the system. The split distribution reflects the occurrence of membrane buckling of either a single‐mode of larger amplitude (Figure 2B, bottom), or of two modes of lower amplitude (Figure 2B, middle). A closer assessment of the CG simulations, compared to the non‐rotating controls, showed that rotation produced a small tilt of the *c*‐ring up relative to the *a* and *b* subunits (Figure S4, Supporting Information). We then explored whether the ATP synthase restrained to the average tilted position but in the absence of rotation induces membrane fluctuations. However, as can be seen in the peak‐to‐peak distance distributions (Figure 2C), enforcing tilt without enforcing rotation results in essentially the same behavior as for the control simulations. We could thus discard the internal tilting effect on its own as an alternative mechanism for the enhanced fluctuations.

Further, we detected the presence of protein‐enriched regions at specific membrane sites with higher curvature under rotating conditions (Figure 2B; Movies S1 and S2, Supporting Information). To quantitatively characterize the curvature sorting of F_o_ proteins, we monitored the average curvature of membrane patches in the vicinity and in the absence of proteins (Figure 2D). Under torque, proteins exhibited a preference for positive membrane curvatures (positive curvature is defined here so that the F_1_ domain would point up), whereas membrane regions devoid of proteins tended to have negative curvatures. The corresponding mean radii of positive curvature in the proximity of the rotating proteins were ≈ 57 and ≈ 73 nm in the synthesis and hydrolysis mode, respectively. These values agree with the experimental range of curvature explored in tube pulling experiments, where the sorting ratio was measured for tubes with a radius of 50 ± 20 nm (Figure 1D). A similar trend was found for the passive proteins but the explored curvatures were significantly smaller and with values close to zero. This is consistent with the enhanced membrane fluctuations promoted by protein rotation (Figure 2B; Movies S1 and S2, Supporting Information), where larger amplitudes produce intrinsically higher curvatures at a constant membrane area.

### 2.3 Rotation‐Induced Protein‐Protein Interaction

**Figure 3:**
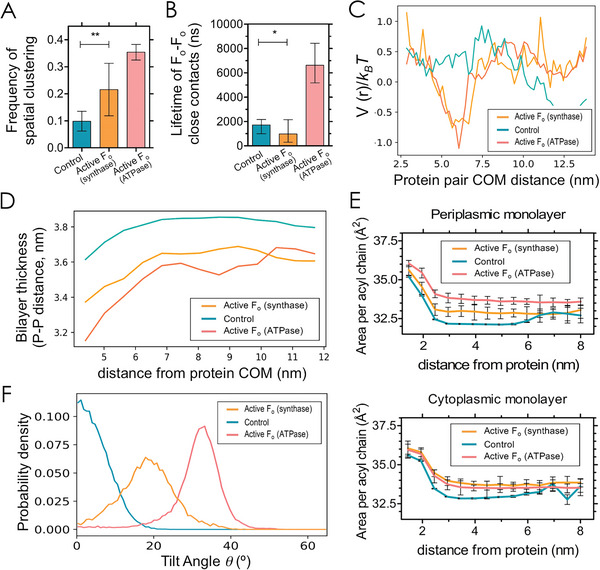
Rotating and passive F_o_ domains generate distinct profiles of lipid packing and have different tendencies to cluster. A) Frequency of spatial clustering of F_o_ domains (within a 2 nm threshold distance of one another). Error bars represent the 95% confidence interval, estimated using a bootstrap; mean comparison (*p*‐value < 0.01) was done with a *t*‐test over sub‐averages of blocks longer than the autocorrelation time. B) Self‐weighted lifetimes of close contacts (within 0.7 nm of one another) between pairs of F_o_ domains (*i.e*., average of the lifetime of each F_o_–F_o_ close contact event, weighted by each event's lifetime). Error bars represent the 95% confidence interval, estimated using a bootstrap method over the weighted lifetimes. Mean comparison (*p*‐value < 0.01) was done with an independent Welsh *t*‐test with Satterthwaite degrees of freedom. C) Protein‐protein interaction potential as a function of the distance between two monomeric ATP synthases. The effective interaction potential was calculated from the relative distances between pairwise proteins (see Methods for details). D) Thickness of bilayers measured by the inter‐leaflet distance between phosphate beads (P–P distance) as a function of the distance to the centre of mass (COM) of F_o_ domain. E) Area per acyl chain as a function of the distance from an active or passive F_o_ domain in the periplasmic (top) and cytoplasmic (bottom) monolayers. F) Tilt angle probability distribution of F_o_ proteins.

A remarkable consequence for the active enrichment of F_o_ domains in curved regions was the apparent clustering of proteins compared to the controls (Movies S1‐S3, Supporting Information). We measured the spatial clustering of F_o_ domains at two levels: i) the loosely bound case, where proteins were counted as aggregated if at an inter‐protein distance below 2 nm, and ii) the close contact case, considering configurations with inter‐protein distances below 0.7 nm (where contacts occur without intervening lipid molecules). We found the formation of loosely bound aggregates to be increased in the active case (Figure S5, Supporting Information), with interactions involving not only proteins that start as first neighbours, but also non‐consecutive proteins. In non‐rotating conditions, such F_o_‐F_o_ clustering occurred less frequently, and only involving proteins that already started off as neighbours (**Figure** **3**A; Figure S5, Supporting Information). In the absence of rotational movement, close F_o_‐F_o_ contacts (at distances below 0.7 nm) were characterized by a time‐weighted average lifetime closer to 2 µs (Figure 3B; Figure S5, Supporting Information). In contrast, proteins in the hydrolysis mode tended to remain in one another's vicinity for longer, with significantly higher lifetimes close to 6 µs. Conversely, close protein contacts were inhibited in the synthesis mode; contact lifetime distribution was polydisperse, mainly containing sub‐µs contact times, but longer contacts were also measured, yielding a time‐weighted average lifetime of 1 µs (Figure 3B; Figure S5, Supporting Information). Whereas the synthesis rotation of the *c*‐ring favors the neighboring between F_o_ proteins, the hydrolysis mode promotes F_o_ sorting into common membrane regions, but discourages their close contact. In this case, the slow diffusion of ATP synthase resulting from Brownian motion occurs over time scales possibly incompatible with the quick, reversible contacts observed for the synthesis mode. Active membrane undulations may be promoting a diffusional acceleration, acting simultaneously as a driver of protein clustering and as the disrupting force of close F_o_‐F_o_ contacts.

To further characterize the self‐association of F_o_ proteins under rotating conditions we determined the effective interaction potential from the probability distribution of relative distances between pairwise proteins (see Methods). The obtained results demonstrate that the two rotating proteins are clustered together by a weak, short‐range and membrane‐mediated potential (Figure 3C). The rotating F_o_‐F_o_ interaction, unlike the non‐rotating case, has a free‐energy minimum of ≈ 1.5 *k_B_T* at a center‐to‐center distance of ≈ 6 nm. This explains the tendency for the loosely bound configurations, since at this distance the protein association does not involve close contacts. This distance also matches the range of inter‐protein distances when enhanced fluctuations are visible (Figure 2B). At closer distances, the energy potential rises smoothly as proteins begin to repel each other, likely due to the membrane bending. In contrast, non‐rotating F_o_ proteins display a non‐interacting potential all over the relative distances. Thus, rotation promotes reversible F_o_ dimers and enhances membrane fluctuations.

### 2.4 Clustering and Curvature Sensing

At small length scales, where thermal fluctuations cannot be neglected, membrane‐mediated interactions originate from local perturbations in the lipid membrane caused by protein activity. Among those perturbations, hydrophobic mismatch between the protein and the bilayer thickness can result into attractive and short‐range interaction between transmembrane proteins with a typical decay length of ≈1 nm,^[^ ^31^, ^32^ ^]^ Attraction arises from the decrease in the amount of deformed interface after protein clustering. To assess how rotation affects the hydrophobic mismatch between *c*‐rings and lipids, we measured the thickness profile of the bilayer as a function of the distance to the proteins as computed by the inter‐leaflet distance between phosphate beads (P–P distance) (Figure 3D). In the passive case, the inclusion of *c*‐ring causes a slight (≈2 Å) the thinning of the first layer of lipids (up to 1 nm). Then, the thickness of the lipid bilayer, *d*, then increases at farther distances from F_o_ proteins until it reaches typical values for POPC lipids, at *d* ≈ 3.9 nm.^[^ ^33^ ^]^ This small variation in the thickness at such a short distance is likely to have little or no effect on protein aggregation,^[^ ^34^ ^]^ in agreement with the non‐interacting potential of non‐rotating proteins (Figure 3C). Conversely, under rotating conditions we found a systematic thinning of the lipid bilayer up to ≈ 7 nm away from the F_o_ proteins. For this stronger membrane thinning (down to *d* ≈ 3.2 nm), hydrophobic mismatch might sustain the attractive interaction between F_o_ proteins.

In the framework of linear elasticity, lipid bilayers can be simply modelled by two thin fluid layers where the bending stiffness, κ, the stretching modulus, *K*, the surface viscosity, η, and the thickness of the bilayer, *d*, are highly correlated to the mean molecular area of the constituent lipids.^[^ ^35^ ^]^ In consequence, a change in membrane curvature or hydrophobic mismatch can be balanced by the chain packing energy and vice versa.^[^ ^36^ ^]^ To get deeper into the mechanism of the rotation‐mediated hydrophobic mismatch we computed the area per acyl chain of POPC lipids, *a*, as a function of the proximity distance from the F_o_ proteins both in the upper (periplasmic) and bottom (cytoplasmic) leaflets (Figure 3E). In all cases, the first layer lipids (up to 2 nm) presented larger areas per acyl chain, which smoothly decreased to constant values at farther distances from F_o_ proteins. The larger areas of acyl chains at the close vicinity of the proteins can be explained by a local perturbation of proteins within the embedding membrane. However, we found a systematic expansion of the lipid chains (≈ 1 %) under rotating conditions up to ≈ 6 nm away from the F_o_ proteins. Beyond this distance, the rotational movement of the *c*‐ring did not affect the lipid packing and the acyl per chain areas reached typical values for unsaturated phosphoplipids, a≈33 nm^2^.^[^ ^37^ ^]^ As expanded monolayers present lower membrane thickness and mechanical moduli described by different scaling laws,^[^ ^35^ ^]^ the local expansion produced by the rotation of the *c*‐ring renders the membrane more deformable, thus presenting thinner thickness and larger amplitude undulations, which are sustained by a lower effective bending modulus.^[^ ^16^ ^]^

**Figure 4:**
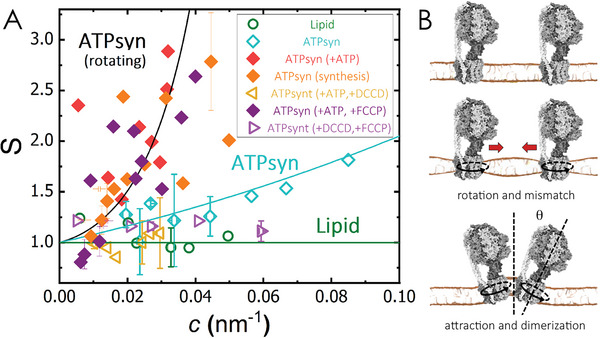
A) Protein sorting as a function of the lipid tube curvature, *c*, for GUVs containing a red fluorescent lipid (control), ATP synthase (ATPsyn), ATPsyn with ATP, ATPsyn in the synthesis mode, ATPsyn with ATP and DCCD, ATPsyn with ATP and FCCP and ATPsyn with DCCD and FCCP. Points correspond to binned sorting values. Solid lines are guides to the eye. B) Graphical model for curvature sorting of rotating ATP synthases. Top: proteins under passive conditions (no rotation) show no mismatch on lipid surroundings. Middle: upon rotation, ATP synthases promote a lipid‐protein mismatch, which produces a membrane‐mediated attractive interaction between proteins. Bottom: at close contact, proteins arrange into a conic‐like conformation with a wedged angle, φ ≈ 2θ, yielding their sorting into curved regions of the lipid membrane.

An apparent consequence of protein clustering and enhanced fluctuations is the change in the orientation of F_o_ proteins. A tilt angle was defined as the angle between the simulation box's *z*‐axis and the protein axis. Whereas the tilt angle of free proteins was close to 0°, clustered proteins in curved regions displayed tilt angles ≈ 20°–30° (Figure 3F). These tilts are a consequence of the preference of the rotating F_o_ dimers for curving membrane regions, yet with individual monomers rarely locating at the crest of the undulations but mostly at their slopes (see the examples in Figure 2D). Such configurations seem to be stabilised by cluster formation. As we do not simulate the bulky F_1_ domain, it is likely that tilt angles might be even more pronounced in in vitro experiments, leading to a conic‐like arrangement of transient dimers. As curvature protein sorting mainly results from a direct coupling of the protein shape and the membrane curvature, the measured curvature sorting of ATP synthase upon ATP incubation (Figure 1D) might be then compatible with the observed protein clustering produced under rotating conditions. To estimate the effective spontaneous curvature of ATP synthase clusters leading to active curvature sorting, the sorting parameter, *S*, was plotted as a function of the curvature of the filament (**Figure** **4**A). Note that an *e*‐fold change in protein sorting depends both on the spontaneous curvature and the protein area through $c_{shape}=\frac{k_{B}T}{A_{p}\kappa_{l}c_{p}}.$ ^[^ ^38^ ^]^ For POPC, the spontaneous curvature is negligible, $c_{POPC}\approx 0$ nm^−1^, and S=1 independently of the curvature of the tube. The absence of significant curvature sorting for passive ATP synthase at low curvatures $\left(c<0.1\;{\mathrm{nm}}^{-1}\right)$ relies on the quite cylindrical profile of the protein‐lipid interface. The wedge angle of ATP synthase at the protein–lipid interface is φ_*ATPsyn*_ ≈ 9° (Figure 1A). From the protein radius $\left(R_{ATPsyn}\approx 3\;\mathrm{nm}\right)$, the spontaneous curvature of ATP synthase is estimated through $c_{P}\approx \frac{\sin(\varphi_{P}/2)}{R_{P}}$, which provides $c_{ATPsyn}\approx 0.03\;{\mathrm{nm}}^{-1}$. As a result, symmetric and large ATP synthase $\left(A_{ATPsyn}\approx 25.6\;{\mathrm{nm}}^{2};c_{ATPsyn}\approx 0.03\;{\mathrm{nm}}^{-1}\right)$ undergoes significant sorting only at high curvatures ($c_{shape}^{ATP\;synthase}\geq 0.1\;{\mathrm{nm}}^{-1}$, with $\kappa_{POPC}=10\;k_{B}T$ ^[^ ^39^ ^]^), as experimentally observed. In contrast, the higher effective spontaneous curvature of clustered ATP synthase dimers might couple with thicker lipid tubes and ultimately be responsible for the curvature sorting. Rotating ATP synthases displayed an *e*‐fold change in protein sorting at much lower curvatures, $c_{shape}^{cluster}\approx 0.03\;{\mathrm{nm}}^{-1}$, which leads to a spontaneous curvature of clustered proteins of $c_{ATPsyn}^{cluster}\approx 0.07\;{\mathrm{nm}}^{-1}$ (with $A_{ATPsyn}^{cluster}\approx 50\;{\mathrm{nm}}^{2}$) and a wedge angle of φ_*cluster*_ =  2θ ≈ 60° (for $R_{ATPsyn}^{cluster}\approx {\left(A_{ATPsyn}^{cluster}\right)}^{\frac{1}{2}}\approx 7\;\mathrm{nm}$), as observed in CG simulations (Figure 3F).

In summary, both experiments and simulations picture an active curvature sorting of rotating ATP synthases by disrupting the lipid packing and thus causing both the membrane thinning and softening of the annular lipids surrounding them.^[^ ^40^ ^]^ As a result, rotation of the *c*‐ring produces a weak, short‐range and membrane‐mediated potential that promotes the formation of reversible and conic‐like transient dimers (Figure 4B).

## 3 Discussion

Our experiments demonstrate that the rotary movement of *c*‐ring promotes the active curvature sorting of monomeric ATP synthases. Coarse‐grained simulations provide the underlying mechanism for curvature sorting, where rotation of the *c*‐ring causes a hydrophobic mismatch at the lipid‐protein interface, thus promoting protein‐protein transient contacts through a membrane‐mediated attractive interaction. The transient ATP synthase dimers display tilt angles of 20–30° that likely cause their curvature sorting into lipid filaments.

To arrive at these conclusions we used simplified systems where membranes were composed of a single lipid but many factors might modulate this association process in a physiological environment. Real biomembranes contain hundreds of lipid species that might interfere with the protein‐protein interaction leading to curvature sorting. Also, phospholipids are not evenly distributed across the lipid leaflets of membranes but rather arranged asymmetrically.^[^ ^41^ ^]^ Moreover, specific interactions with particular lipids might change the lateral distribution of the reconstituted proteins.^[^ ^42^ ^]^ Furthermore, cardiolipin shows a strong interaction with ATP synthase,^[^ ^43^, ^44^ ^]^ and prone to curvature sensing.^[^ ^26^ ^]^ To discard this enhancing effect, we used GUVs composed of only one lipid species, POPC, which is known to be absent in bacterial membranes.^[^ ^45^ ^]^ Moreover, a high protein‐to‐phospholipid ratio characterizes ATP synthase‐containing membranes. In particular, both the inner mitochondrial membrane and the *E.coli* inner membrane exhibit a high protein level up to 80 % w/w.^[^ ^46^, ^47^ ^]^ Despite high protein content in those membranes, the short‐range interactions between rotating ATP synthases will favor the formation of transient dimers of nearby proteins.

As mitochondrial ATP synthases are mostly found in the rim of *cristae* forming dimers, it is therefore tempting to suggest a new role for the *c*‐ring rotation in the *cristae* biogenesis and maintenance.^[^ ^12^ ^]^ Mitochondrial ATP synthases are assembled as monomers within *cristae* ^[^ ^48^ ^]^ and would dimerize at the tip of *cristae* via specific interaction of two adjacent *b* subunits and the participation of subunits *e* and *g*, among others.^[^ ^8^, ^49^, ^50^ ^]^ However, it is not known if dimers are assembled previously to their clustering into the tip of *cristae* or if the accumulation of monomeric proteins into curved regions favor their stabilization through the specific interaction provided by the different F_o_ subunits. Clues into this behavior include the fact that monomeric ATP synthases can also be found at the inner boundary membranes, likely due to protein diffusion from *cristae* or to the protein exchange during mitochondrial dynamics.^[^ ^51^ ^]^

The width of *cristae* membranes depends on the organism, the cell type, and the metabolic and health status of mitochondria.^[^ ^52^ ^]^ In general, *cristae* have an approximate curvature radius between 10 and 15 nm.^[^ ^53^ ^]^ We did not experimentally achieve such curvatures under rotating conditions, but proteins exhibited a degree of spontaneous sorting (in the absence of rotation, S=1.5. See Figure 4A) at moderate curvatures (c>0.06). Upon extrapolation of our data from active conditions in the presence of ATP, the sorting parameter would reach values as high as S>4). Our results suggest that the rotational movement of the *c*‐ring might be a determinant funnelling factor for the dynamic arrangement of ATP synthases within different membrane regions of *cristae*.

Additionally, ATP synthases are found in bacterial and thylakoid membranes. As bacterial and thylakoid ATP synthases are not furnished with the extra subunits responsible for protein dimerization, they are assumed to appear as monomers in their membrane environments. Those ATP synthases keep their ability to rotate, prompting questions about the protein‐protein contacts and the formation of *cristae*‐like structures in those biomembranes. On one hand, the high internal turgor pressure inside the bacterial cytoplasm (up to 3 MPa for Gram‐positive bacteria) is not conductive to the high excess area required for the formation of tubular structures. However, a non‐uniform localization of ATP synthases in *B. subtilis* has been observed, where ATP synthases were found restricted to irregular and highly dynamic domains within the membrane.^[^ ^54^ ^]^ In addition to the likely lipid‐driven protein segregation through specific protein‐lipid interactions, it will be interesting to determine whether the formation of these protein‐rich domains is correlated with the rotating movement of proteins.

The existence of ATP synthase dimers in chloroplasts has been confirmed for a variety of organisms, including plants and green algae.^[^ ^55^ ^]^ A recent study suggests that up to 15 % of thylakoid ATP synthases appear to be in contact with another ATP synthase.^[^ ^56^ ^]^ These dimers are not stabilized through *f* and *g* subunits, as these are absent in chloroplast ATP synthases. Interestingly, vanadate inhibition of ATPase activity results in depletion of ATP synthase dimers in the thylakoid membranes of *Chlamydomonas reindhartii*.^[^ ^57^ ^]^ This observation suggests a link between ATP synthase rotation and the ability of ATP synthases to dimerize. Contrary to our curvature‐sensing observations, however, ATP synthases in chloroplasts are only found in flat areas and excluded from the margins of thylakoids.^[^ ^58^ ^]^ Such particular organization of proteins may be explained by the singular architecture of these organelles. First, the extreme curvature of the margins may be too acute for a positive sorting, instead promoting a negative sorting. Note that the thylakoid luminal thickness between grana membranes is only 4.5 nm,^[^ ^59^ ^]^ in contrast with the approximately 27 nm in mitochondrial *cristae*.^[^ ^53a^ ^]^ Second, proteins in the thylakoid exist in a quasi‐crystalline, crowded state.^[^ ^60^ ^]^ In such a rigid membrane, hydrophobic mismatch interaction might be even higher^[^ ^34^ ^]^ for protein dimerization but chloroplast ATP synthases might be confined to grana‐end and *stroma lamellae* areas, prevented from reaching the highly curved margins. This can be compounded by specific activity, such as that of the CURT1 proteins that have been reported to stabilize high‐curvature thylakoid regions and to block access to other proteins.^[^ ^61^ ^]^ Interestingly, these observations in bacterial and thylakoid membranes can be interpreted through the lens of the protein‐protein approximations promoted by the *c*‐ring rotation. Deeper investigation, using complex living systems, will hopefully be able to further connect molecular‐level details to biomembrane behavior.

## 4 Conclusions

Monomeric F_1_F_o_‐ATP synthases from *E.coli* were reconstituted in GUVs and their curvature sorting was measured through tether pulling experiments. The curvature‐seeking behavior of ATP synthases was only observed for rotating proteins i.e., under active conditions. The incubation with the rotation inhibitor DCCD prevented the protein sorting into nanometric lipid filaments. Coarse‐grained molecular dynamics simulations showed that the rotation of simulated F_o_ proteins stabilized membrane buckling, where the preference of proteins for high curvature was significantly higher than in controls. Additionally, F_o_ proteins underwent an apparent clustering under torque, with frequent and reversible loose association of proteins. The underlying mechanism of curvature sorting was compatible with a membrane‐mediated protein‐protein interaction. The attractive force between F_o_ domains relies on the hydrophobic mismatch produced at the vicinity of rotating proteins, which produce the formation of transient dimers with a conic‐like arrangement, thus likely causing the curvature sorting in in vitro experiments. Overall, our results suggest a new functional role for the *c*‐ring rotatory movement of ATP synthases in biomembranes.

## 5 Experimental Section

### Chemicals

Potassium chloride (KCl), magnesium chloride (MgCl_2_), sodium chloride (NaCl) glucose, sucrose, tris(hidroximetil)aminometano (Tris), 4‐(2‐hydroxyethyl)−1‐piperazineethanesulfonic acid (HEPES), N,N′‐dicyclohexylcarbodiimide (DCCD), carbonyl cyanide‐4‐(trifluoromethoxy) phenylhydrazone (FCCP), hydroxypyrene‐1,3,6.trisulfonic acid (Pyranine), Adenosine 5′‐triphosphate disodium salt (ATP), β‐casein, bovine serum albumin (BSA), lysine and mineral oil were supplied by Sigma‐Aldrich. n‐Dodecyl‐β‐d‐maltoside (DDM) was purchased from VWR. Phosphate buffer saline (PBS) was provided by GE Healthcare. Polystyrene streptavidin‐coated microspheres (≈10 µm diameter) were purchased from Bangs Laboratories, Inc. Alexa 555‐NHS was acquired from Thermofisher (Molecular Probes). Ultrapure water was produced from Milli‐Q unit (Millipore, conductivity lower than 18 MΩ cm^−1^).

### Lipids

1,2‐dioleoyl‐sn‐glycero‐3‐phosphoethanolamine‐N‐(lissamine rhodamine B sulfonyl) (ammonium salt) (RhPE), 1,2‐palmitoyloleyl‐sn‐glycero‐3‐phosphocholina (POPC) and 1,2‐distearoyl‐sn‐glycero‐3‐phosphoethanolamine‐N‐[biotinyl(polyethylene glycol)−2000] (ammonium salt) (DSPE‐PEG(2000) Biotin) were purchase from Avanti Polar. 1,2‐Dioleoyl‐sn‐glycero‐3‐phosphoethanolamine fluorescently labelled with Atto 488 (DOPE Atto‐488) was purchased from ATTO‐TEC. Lipids were suspended in chloroform at 1 mg mL^−1^ and stored at −20 °C.

### Electroformation of Giant Unilamellar Vesicles (GUVs)

Giant vesicles were prepared using the standard electroformation protocol.^[^ ^62^ ^]^ The fabrication chamber was composed of two 1‐mm spaced conductor indium tin oxide (ITO)‐coated slides (7.5 × 2.5 cm^2^; 15–25 Ω sq^−1^ surface resistivity; Sigma). Briefly, GUVs were prepared by transferring a volume of 10 µL of the POPC solution in chloroform (0.5 mg mL^−1^) onto each ITO slide. Samples were dried at room temperature and rehydrated in buffered solution (25 mM HEPES pH 7.4, 10 mM KCl, 155 mM sucrose) and the electrodes were connected to an AC power supply (10 Hz, 1.1 V; Agilent) for at least 3 h. POPC lipids were supplemented with 1.2 % mol DOPE Atto‐488 and 0.4 % mol DSPE‐PEG(2000) Biotin for pulling experiments. Lipid sorting was also carried out with 1.2 % mol RhPE.

### Protein Purification and Fluorescent Labelling

Bacterial F_1_F_o_–ATP synthase was purified from native *E.coli* MG1655 cytoplasmic membranes as described in^[^ ^63^ ^]^ and fluorescently labelled with fluorescent Alexa 555‐NHS as described in.^[^ ^16^ ^]^ Bacteriorhodopsin from *Halobacterium salinarum* (Sigma Aldrich) was also fluorescently labelled with Alexa 555‐NHS using the same protocol. Labelled proteins were conserved in stock solution (200 mM sucrose, 10 mM KCl, 10 mM Tris HCl pH 8, 0.5 mM DDM) at −80 °C.

### Protein Reconstitution

Protein reconstitution into GUVs was performed by mild detergent treatment with DDM.^[^ ^64^, ^65^ ^]^ Briefly, 0.4‐1 µL of protein stock solution (≈ 0.2 mg mL^−1^) was diluted in with 10 µL of GUV solution and incubated for 45 min at room temperature. The DDM concentration was then below its critical micelle concentration (cmc_DDM_ = 0.17 mM), promoting protein incorporation into the membranous environment.

### Tube Pulling Assay

The micromanipulation device consisted of two homemade micromanipulators, using pipette holders (Narishige) and micrometre screws with < 1 µm accuracy in the three *x*, *y*, and *z* axes mounted on vertical platforms (ThorLabs).^[^ ^66^ ^]^ Borosilicate capillaries (0.5 mm ID and 1 mm OD, World Precision Instrument) were pulled (PC‐100, Narishige) and forged (MFG‐5, Microdata Instruments) to form tips with 5–12 µm internal diameter. The micropipettes were connected through the pipette holders to water tanks and aspiration was controlled by hydrostatic pressure up to 3 kPa with 20 mPa resolution steps. The micromanipulation chamber was made up of two 4‐mm spaced coverslides with a volume of ≈ 200 µL, allowing pipettes to enter the solution. The whole system was mounted on a Nikon Ti‐E inverted microscope equipped with a Nikon C2 confocal scanning confocal module, 488‐nm and 561‐nm continuous lasers (Sapphire), Plan Apo 100× NA 1.45 oil immersion objective and PL Apo 40× water objective (Nikon).

Prior to any operation, both the pipettes and the chamber were incubated with BSA (1 mg mL^−1^) for 5 min at room temperature to inhibit strong adhesion of vesicles to the bottom of the observation chamber. After washing, the chamber was filled with 200 mM glucose solution or salt buffer (15 mM HEPES pH 7.4, 5 mM MgCl_2_, 55 mM NaCl, 100 mM glucose), 10–20 µL of proteo‐GUV solution and 1–2 µL of a biotinylated silica beads (1:10 dilution of manufacturer solution). For active conditions, ATP (20 mM ATP, 20 mM potassium phosphate buffer pH 7.2, 20 mM MgCl_2_; ATP 1 mM final concentration) was added in the chamber buffer before starting the micromanipulation experiment. For F_o_ inhibition experiments, the same external media used for active conditions was complemented with DCCD (1 mM final concentration, see Supporting Information for details). For dissipating experiments under active conditions in the presence of ATP and FCCP, the protonophore was included in the external medium (20 µM final concentration). For the proton passive translocation experiments with FCCP and DCCD, ATP synthase GUVs were diluted in acidic buffer (15 mM HEPES pH 6.8, 5 mM MgCl_2_, 55 mM NaCl, 100 mM glucose). In the synthesis mode, ATP synthase GUVs were diluted in synthesis buffer containing 15 mM HEPES pH 7.4, 5 mM MgCl_2_, 55 mM KCl, 100 mM glucose, 10 mM Na_2_ADP, 10 mM K_2_HPO_4_ and 10 µM valinomycin. Pulling experiments were performed during 30 minutes after mixing GUVs with external media, a time window where the proteins remained active or inhibited (see Supporting Information for details). BR curvature sorting experiments under passive conditions were performed using the same protocol as for ATP synthase in the absence of ATP whereas BR GUVs under illumination were diluted in 15 mM HEPES pH 7.4, 5 mM MgCl_2_, 55 mM KCl, 100 mM glucose and valinomycin 10 µM to overcome the retroinhibitory effect of the electrochemical gradient by the proton pumping activity of BR.^[^ ^25^ ^]^ Light‐induced proton transport by BR was performed illuminating the sample for 30 min using a 12‐V, 100‐W halogen lamp with an emission band‐pass filter (360‐460 nm) prior to imaging for protein sorting.

For each pulling experiment, a slightly fluctuating vesicle was selected and held with a micropipette under a minimal suction pressure and a biotinylated silica bead was held with the second pipette under high suction pressure. The microaspirated GUV and the bead were brought into contact and a membrane tube was pulled from the GUV upon separation of the bead. For control experiments in the absence of proteins, the tube radius was measured with several back and forth pulling movements. As the surface and volume of GUVs remain constant during pulling experiments, the tube radius, $r_{t}$, was obtained from the relative changes of the aspirated protrusion, $L_{p}$, with the tube length, $L_{t}$, up to ≈ 160 µm:

(2) $$r_{t}=\;-\left(\frac{dL_{P}}{dL_{t}}\right)\left(1-\frac{R_{p}}{R_{V}}\right)R_{P}$$

where $R_{p}$ is the pipette radius and $R_{V}$ is the vesicle radius.^[^ ^15^ ^]^ After each pulling in and out step, a waiting time of 30 seconds was set up to allow the tube composition to equilibrate. Also, a calibration factor, *F*, was obtained through $r_{t}=F\times \left(I_{tube}^{lip}/I_{GUV}^{lip}\right)$. This allowed to measure $r_{t}$ directly from the lipid fluorescence channel in proteo‐GUVs.^[^ ^38^ ^]^ Finally, the protein sorting was obtained by comparing the lipid and the protein fluorescence intensity of the membrane tube to that of the GUV following the (Equation 1) of the main text.^[^ ^13^ ^]^ The sorting ratio was averaged for tubes with radii, $r_{t}=50\pm 20\;\mathrm{nm}$. The fluorescent intensities were obtained with a homemade MATLAB script. More details in Supporting Archive 1.

### Pyranine Assays for Testing Protein Activity after Reconstitution in GUVs

The proton transmembrane translocation activity of ATP synthase and BR was assessed using the pH–sensitive probe pyranine as a reporter of the luminal acidification of proteo‐GUVs^[^ ^64^, ^67^ ^]^ (see Figure S6, Supporting Information). Pyranine was encapsulated at 150 µM into GUVS prior to protein reconstitution and the luminal acidification was deduced from the decrease of the fluorescence intensity of the probe excited at *λ*_***ex***_  =  488 nm as reported previously.^[^ ^16^ ^]^

### Simulation Setup

Martini 3 version parameters were used for the protein, lipid, and solvent.^[^ ^27^ ^]^ The atomistic model of the transmembrane domain of the *E. coli* ATP synthase (F_o_) (PDB ID 6vwk)^[^ ^68^ ^]^ was converted into a coarse‐grained structure using the *vermouth‐martinize* python script^[^ ^69^ ^]^ with default elastic network parameters: elastic bonds between backbone particles within 0.9 nm of one another, with force constant 500 kJ mol^−1^ nm^−2^. To allow for independent rotation, the *c*‐ring was modelled as a single elastic‐network‐connected unit, and *a* and *b* subunits as another unit. The proton‐carrying *c*‐ring Asp residues that sit exposed to the membrane core were all modelled in their protonated state. Four F_o_ domains were inserted into a bilayer consisting of 1712 molecules of POPC, using the *insane* script.^[^ ^70^ ^]^ The membrane was set up with a large aspect ratio so that any undulations would occur essentially over one dimension. The system was solvated with a 150 mM NaCl water solution, and counterions were added until neutrality.

### Molecular Dynamics Simulations

Simulations were run using the GROMACS simulation package version 2021.1^[^ ^71^ ^]^ patched with PLUMED version 2.7.1.^[^ ^72^ ^]^ Structures equilibrated in the NPT ensemble (using a semi‐isotropic Parrinello‐Rahman barostat with a 12 ps time constant) were used as the starting point for molecular dynamics simulations of four replicates of at least 5 µs. A time‐step of 20 fs was used with a Verlet cut‐off scheme. Temperature was maintained at 300 K using a v‐rescale thermostat coupled separately to the bilayer and the solvent with a time constant of 1 ps. Simulations were run in the NVT ensemble so that any rotation‐induced local membrane expansion would not be masked by barostat action. A reaction‐field Coulombic potential with a 1.1 nm cut‐off radius was used. For Van der Waals interactions, a Lennard‐Jones potential was applied, cut‐off at 1.1 nm and translated by a constant so that the potential was zero at the cut‐off.

To induce the rotation of the *c*‐ring of the F_o_ transmembrane domain relative to the *a* and *b* subunits, a torque was imposed on one of the 10 subunits of the *c*‐ring using PLUMED (via a 400 kJ mol^−1^ rad^−1^ force applied to the rotation of the *c*‐ring relative to the *a* and *b* subunits; Figure S3 (Supporting Information) shows rotation profiles at lower and higher torques). A restraint was added keeping the centre of the *c*‐ring at a constant distance of three points in the *a* subunit, so that the imposition of a torque would not cause the separation of the *c*‐ring from the other F_o_ subunits. Details on the application of torque and restraints were expanded in the Supporting Methods. For ease of reproducibility, the starting structures, topologies, and GROMACS and PLUMED setup files were also all included as a Supporting Archive 2.

In a specific case, instead of a torque, additional harmonic restraints were applied to induce tilting of the *c*‐ring relative to the *a* subunit. These restraints were applied as an elastic network on all backbone–backbone distances between the *c*‐ring and the *a* subunit under 0.9 nm. The reference for these distances was the average tilted conformation the F_o_ units adopt when under torque.

### Simulation Analysis and Visualization

Analysis of the trajectories was performed using in‐house analysis scripts, with extensive use of the NumPy,^[^ ^73^ ^]^ SciPy,^[^ ^74^ ^]^ and MDAnalysis Python packages.^[^ ^75^, ^76^ ^]^ The rotational velocity of the *c*‐ring was assessed by measuring the mean rotation (in radians) per unit time of all proteins divided by 2π. For the analysis of the curvature preference of the F_o_ protein, the average *z*‐coordinate profile inscribed by the lipid phosphate groups along the *x*‐axis was obtained, then sliced into 30 fragments of equivalent size. The intersection points of the slices with the profile were used as the vertices of the triangles from which the local curvature of the bilayer was obtained from the circumradius of the circle that circumscribes each triangle (Figure S7, Supporting Information for details). The fluctuation of the membrane in the active and passive modes was explored by monitoring the distribution of the peak‐to‐peak (maxima−minima) distances of the lipid phosphate *z*‐coordinates throughout the trajectory. For the average protein‐protein contact lifetimes, a cut‐off distance of 0.7 nm was defined. In contrast, for the time‐averaged inter‐F_o_ approximations, the distance cut‐off was of 20 *Å*. To obtain the interaction free‐energy between pairs of proteins as a function of distance, the probability distribution of pair protein distances, p(l) was calculated for the second half of the simulations. Assuming a Boltzmann distribution, the effective potential V(l) can be deduced through:

(3) $$V\;\left(l\right)=\;-k_{B}T\ln\left(p\left(l\right)\right)-\ln Z$$

with

(4) $$Z\;=\int_{0}^{\infty }e^{\frac{-V\left(l\right)}{k_{B}T}}2\pi l\;dl$$

The thickness profile of the bilayer was calculated as follows: first, the distribution of the *z*‐coordinates of the lipid phosphates per monolayer was obtained as a function of the distance from the center of mass (COM) of the protein. A corrective factor to account for the curvature of the bilayer was applied to the *z*‐axis. The thickness was estimated as the difference between the *z*‐coordinates of the upper and the bottom monolayers. The tilt angle was defined as the angle between the simulation box's *z*‐axis and the vector pointing from B to T (B was the COM of the 8—one per *c*‐subunit of the *c*‐ring—bottom ASN3 backbone beads; T was the COM of the 8 top PRO43 backbone beads of the *c*‐ring). VMD 1.9.3 was used for molecular visualization.^[^ ^77^ ^]^ All scripts were available in Supporting Archive 3.

### Statistical Analysis

Bending moduli and sorting data were tested via ANOVA. Differences between means were assessed using the Tukey Mean Difference test. Statistical significance difference was established as sn (*p*‐value > 0.05), * (*p* ≤ 0.05), **(*p* ≤ 0.01) or *** (*p* ≤ 0.001). The difference between curvature preferences was compared using a *t*‐test for independent samples. In the case of the spatial clustering simulation results, a weighted Welch *t*‐test for independent samples was used. Because data from simulation time‐series were naturally autocorrelated, confidence intervals in means of values measured over frames were estimated by a modified bootstrap approach in which each resampling was done over only N/I samples, where *N* is the total number of samples and *I* the integral of their autocorrelation function.^[^ ^55^ ^]^

## Conflict of Interest

The authors declare no conflict of interest.

## Supporting information

Supporting Information

Supplemental Video 1

Supplemental Video 2

Supplemental Video 3

Supporting Archive 1

Supporting Archive 2

Supporting Archive 3

## Data Availability

The data that support the findings of this study are available from the corresponding author upon reasonable request.

## References

[1] P. D. Boyer , Annu. Rev. Biochem. 1997, 66, 717. 9242922 10.1146/annurev.biochem.66.1.717

[2] A. I. Jonckheere , J. A. Smeitink , R. J. Rodenburg , J Inherit Metab Dis 2012, 35, 211. 21874297 10.1007/s10545-011-9382-9 PMC3278611

[3] P. Mitchell , Biol Rev Camb Philos Soc 1966, 41, 445. 5329743 10.1111/j.1469-185x.1966.tb01501.x

[4] H. Noji , R. Yasuda , M. Yoshida , K. Kinosita , Nature 1997, 386, 299. 9069291 10.1038/386299a0

[5] W. Kuhlbrandt , K. M. Davies , Trends Biochem. Sci. 2016, 41, 106. 26671611 10.1016/j.tibs.2015.10.006

[6] I. Arnold , K. Pfeiffer , W. Neupert , R. A. Stuart , H. Schagger , EMBO J. 1998, 17, 7170. 9857174 10.1093/emboj/17.24.7170 PMC1171063

[7] F. Minauro‐Sanmiguel , S. Wilkens , J. J. Garcia , Proc Natl Acad Sci 2005, 102, 12356. 16105947 10.1073/pnas.0503893102 PMC1194923

[8] K. M. Davies , C. Anselmi , I. Wittig , J. D. Faraldo‐Gomez , W. Kuhlbrandt , Proc Natl Acad Sci U S A 2012, 109, 13602. 22864911 10.1073/pnas.1204593109 PMC3427116

[9] T. B. Blum , A. Hahn , T. Meier , K. M. Davies , W. Kuhlbrandt , Proc Natl Acad Sci 2019, 116, 4250. 30760595 10.1073/pnas.1816556116 PMC6410833

[10] P. Paumard , J. Vaillier , B. Coulary , J. Schaeffer , V. Soubannier , D. M. Mueller , D. Brethes , J. P. di Rago , J. Velours , EMBO J. 2002, 21, 221. 11823415 10.1093/emboj/21.3.221 PMC125827

[11] P. D. Gavin , M. Prescott , S. E. Luff , R. J. Devenish , J. Cell Sci. 2004, 117, 2333. 15126633 10.1242/jcs.01074

[12] V. Almendro‐Vedia , P. Natale , D. Valdivieso Gonzalez , M. P. Lillo , J. L. Aragones , I. Lopez‐Montero , Arch. Biochem. Biophys. 2021, 708, 108939. 34052190 10.1016/j.abb.2021.108939

[13] B. Sorre , A. Callan‐Jones , J. B. Manneville , P. Nassoy , J. F. Joanny , J. Prost , B. Goud , P. Bassereau , Proc Natl Acad Sci U S A 2009, 106, 5622. 19304798 10.1073/pnas.0811243106 PMC2667082

[14] A. Tian , T. Baumgart , Biophys. J. 2009, 96, 2676. 19348750 10.1016/j.bpj.2008.11.067 PMC2711293

[15] L. Bo , R. E. Waugh , Biophys. J. 1989, 55, 509. 2930831 10.1016/S0006-3495(89)82844-9 PMC1330504

[16] V. G. Almendro‐Vedia , P. Natale , M. Mell , S. Bonneau , F. Monroy , F. Joubert , I. Lopez‐Montero , Proc Natl Acad Sci U S A 2017, 114, 11291. 29073046 10.1073/pnas.1701207114 PMC5664490

[17] J. Hermolin , R. H. Fillingame , J. Biol. Chem. 1989, 264, 3896. 2521856

[18] M. Toei , H. Noji , J. Biol. Chem. 2013, 288, 25717. 23893417 10.1074/jbc.M113.482455 PMC3764779

[19] K. Nishio , A. Iwamoto‐Kihara , A. Yamamoto , Y. Wada , M. Futai , Proc Natl Acad Sci U S A 2002, 99, 13448. 12357031 10.1073/pnas.202149599 PMC129693

[20] M. Yoshida , W. S. Allison , J. Biol. Chem. 1983, 258, 14407. 6227624

[21] R. Lipowsky , Faraday Discuss. 2013, 161, 305. 23805747 10.1039/c2fd20105d

[22] a) N. Khalifat , N. Puff , S. Bonneau , J. B. Fournier , M. I. Angelova , Biophys. J. 2008, 95, 4924; 18689447 10.1529/biophysj.108.136077 PMC2576396

[23] N. Patil , S. Bonneau , F. Joubert , A. F. Bitbol , H. Berthoumieux , Phys. Rev. E 2020, 102. 10.1103/PhysRevE.102.022401 32942462

[24] M. Strauss , G. Hofhaus , R. R. Schroeder , W. Kuhlbrandt , EMBO J. 2008, 27, 1154. 18323778 10.1038/emboj.2008.35 PMC2323265

[25] M. Seigneuret , J. L. Rigaud , Biochemistry 1986, 25, 6723.

[26] E. Beltran‐Heredia , F. C. Tsai , S. Salinas‐Almaguer , F. J. Cao , P. Bassereau , F. Monroy , Commun Biol 2019, 2, 225. 31240263 10.1038/s42003-019-0471-x PMC6586900

[27] P. C. T. Souza , R. Alessandri , J. Barnoud , S. Thallmair , I. Faustino , F. Grunewald , I. Patmanidis , H. Abdizadeh , B. M. H. Bruininks , T. A. Wassenaar , P. C. Kroon , J. Melcr , V. Nieto , V. Corradi , H. M. Khan , J. Domanski , M. Javanainen , H. Martinez‐Seara , N. Reuter , R. B. Best , I. Vattulainen , L. Monticelli , X. Periole , D. P. Tieleman , A. H. de Vries , S. J. Marrink , Nat. Methods 2021, 18, 382. 33782607 10.1038/s41592-021-01098-3 PMC12554258

[28] a) K. Okazaki , G. Hummer , Proc Natl Acad Sci U S A 2013, 110, 16468. 24062450 10.1073/pnas.1305497110 PMC3799341

[29] R. Yasuda , H. Noji , M. Yoshida , K. Kinosita , H. Itoh , Nature 2001, 410, 898. 11309608 10.1038/35073513

[30] T. Betz , C. Sykes , Soft Matter 2012, 8, 5317.

[31] T. R. Weikl , M. M. Kozlov , W. Helfrich , Phys. Rev. E 1998, 57, 6988.

[32] J. A. Killian , Biochimica Et Biophysica Acta‐Reviews on Biomembranes 1998, 1376, 401. 10.1016/s0304-4157(98)00017-3 9805000

[33] N. Kucerka , S. Tristram‐Nagle , J. F. Nagle , J. Membr. Biol. 2005, 208, 193. 16604469 10.1007/s00232-005-7006-8

[34] R. Phillips , T. Ursell , P. Wiggins , P. Sens , Nature 2009, 459, 379. 19458714 10.1038/nature08147 PMC3169427

[35] E. G. Kelley , P. D. Butler , R. Ashkar , R. Bradbury , M. Nagao , Proc Natl Acad Sci U S A 2020, 117, 23365. 32883879 10.1073/pnas.2008789117 PMC7519290

[36] M. F. Brown , Biochemistry 2012, 51, 9782. 23163284 10.1021/bi301332v PMC5176250

[37] N. Kucerka , Y. F. Liu , N. J. Chu , H. I. Petrache , S. T. Tristram‐Nagle , J. F. Nagle , Biophys. J. 2005, 88, 2626. 15665131 10.1529/biophysj.104.056606 PMC1305359

[38] S. Aimon , A. Callan‐Jones , A. Berthaud , M. Pinot , G. E. Toombes , P. Bassereau , Dev. Cell 2014, 28, 212. 24480645 10.1016/j.devcel.2013.12.012 PMC3954780

[39] G. Niggemann , M. Kummrow , W. Helfrich , J. de Physique II 1995, 5, 413.

[40] M. Fosnaric , A. Iglic , S. May , Phys Rev E Stat Nonlin Soft Matter Phys 2006, 74, 051503. 17279913 10.1103/PhysRevE.74.051503

[41] M. Seigneuret , P. F. Devaux , Proceed. of the National Academy of Sci. of the United States of America‐Bio. Sci. 1984, 81, 3751. 10.1073/pnas.81.12.3751 PMC345297 6587389

[42] A. G. Lee , Biochim. Biophys. Acta 2004, 1666, 62. 15519309 10.1016/j.bbamem.2004.05.012

[43] A. L. Duncan , A. J. Robinson , J. E. Walker , Proc Natl Acad Sci U S A 2016, 113, 8687. 27382158 10.1073/pnas.1608396113 PMC4978264

[44] R. A. Corey , W. L. Song , A. L. Duncan , T. B. Ansell , M. S. P. Sansom , P. J. Stansfeld , Sci. Adv. 2021, 7. 10.1126/sciadv.abh2217 PMC8378812 34417182

[45] C. Sohlenkamp , O. Geiger , FEMS Microbiol. Rev. 2016, 40, 133. 25862689 10.1093/femsre/fuv008

[46] G. Daum , J. E. Vance , Progress in Lipid Research 1997, 36, 103. 9624424 10.1016/s0163-7827(97)00006-4

[47] G. Guidotti , Annu. Rev. Biochem. 1972, 41, 731. 4263713 10.1146/annurev.bi.41.070172.003503

[48] H. Bahri , J. Buratto , M. Rojo , J. P. Dompierre , B. Salin , C. Blancard , S. Cuvellier , M. Rose , A. Ben Ammar Elgaaied , E. Tetaud , J. P. di Rago , A. Devin , S. Duvezin‐Caubet , Biochim Biophys Acta Mol Cell Res 2021, 1868, 118942. 33359711 10.1016/j.bbamcr.2020.118942

[49] J. Habersetzer , I. Larrieu , M. Priault , B. Salin , R. Rossignol , D. Brethes , P. Paumard , PLoS One 2013, 8, e75429. 24098383 10.1371/journal.pone.0075429 PMC3788808

[50] K. Wagner , I. Perschil , C. D. Fichter , M. van der Laan , Mol. Biol. Cell 2010, 21, 1494. 20219971 10.1091/mbc.E09-12-1023 PMC2861609

[51] F. Vogel , C. Bornhovd , W. Neupert , A. S. Reichert , J. Cell Biol. 2006, 175, 237. 17043137 10.1083/jcb.200605138 PMC2064565

[52] C. A. Mannella , Bba‐Mol. Basis Dis. 2006, 1762, 140.

[53] a) N. Ikon , R. O. Ryan , Bba‐Biomembranes 2017, 1859, 1156; 28336315 10.1016/j.bbamem.2017.03.013 PMC5426559

[54] A. S. Johnson , S. van Horck , P. J. Lewis , Microbiology (Reading) 2004, 150, 2815. 15347741 10.1099/mic.0.27223-0

[55] A. Minges , G. Groth , Adv. Bot. Res. 2020, 96, 27.

[56] B. Daum , D. Nicastro , J. A. Il , J. R. McIntosh , W. Kuhlbrandt , Plant Cell 2010, 22, 1299. 20388855 10.1105/tpc.109.071431 PMC2879734

[57] a) H. Schwasmann , S. Rexroth , J. M. zu Tittingdorf , F. Krause , N. H. Reifschneider , N. A. Dencher , H. Seelert , Bba‐Bioenergetics 2006, 321;17140678

[58] M. Pribil , M. Labs , D. Leister , J. Exp. Bot. 2014, 65, 1955. 24622954 10.1093/jxb/eru090

[59] B. Daum , W. Kuhlbrandt , J. Exp. Bot. 2011, 62, 2393. 21441405 10.1093/jxb/err034

[60] C. Wilhelm , R. Goss , G. Garab , J. Plant Physiol. 2020, 252, 153246. 32777580 10.1016/j.jplph.2020.153246

[61] U. Armbruster , M. Labs , M. Pribil , S. Viola , W. T. Xu , M. Scharfenberg , A. P. Hertle , U. Rojahn , P. E. Jensen , F. Rappaport , P. Joliot , P. Dormann , G. Wanner , D. Leister , Plant Cell 2013, 25, 2661. 23839788 10.1105/tpc.113.113118 PMC3753390

[62] L. Mathivet , S. Cribier , P. F. Devaux , Biophys. J. 1996, 70, 1112. 8785271 10.1016/S0006-3495(96)79693-5 PMC1225041

[63] O. Gutierrez‐Sanz , P. Natale , I. Marquez , M. C. Marques , S. Zacarias , M. Pita , I. A. Pereira , I. Lopez‐Montero , A. L. De Lacey , M. Velez , Angew Chem Int Ed Engl 2016, 55, 6216. 26991333 10.1002/anie.201600752 PMC5132028

[64] M. Dezi , A. Di Cicco , P. Bassereau , D. Levy , Proc Natl Acad Sci U S A 2013, 110, 7276. 23589883 10.1073/pnas.1303857110 PMC3645586

[65] J. Sjoholm , J. Bergstrand , T. Nilsson , R. Sachl , C. V. Ballmoos , J. Widengren , P. Brzezinski , Sci. Rep. 2017, 7, 2926. 28592883 10.1038/s41598-017-02836-4 PMC5462737

[66] E. Evans , W. Rawicz , Phys. Rev. Lett. 1990, 64, 2094. 10041575 10.1103/PhysRevLett.64.2094

[67] M. Seigneuret , J.‐L. Rigaud , FEBS Lett. 1985, 188, 101.

[68] M. Sobti , J. L. Walshe , D. Wu , R. Ishmukhametov , Y. C. Zeng , C. V. Robinson , R. M. Berry , A. G. Stewart , Nat. Commun. 2020, 11, 2615. 32457314 10.1038/s41467-020-16387-2 PMC7251095

[69] P. Kroon , Thesis fully internal (DIV), University of Groningen, 2020.

[70] T. A. Wassenaar , H. I. Ingolfsson , R. A. Bockmann , D. P. Tieleman , S. J. Marrink , J. Chem. Theory Comput. 2015, 11, 2144. 26574417 10.1021/acs.jctc.5b00209

[71] M. J. Abraham , T. Murtola , R. Schulz , S. Páll , J. C. Smith , B. Hess , E. Lindahl , SoftwareX 2015, 2, 19.

[72] G. A. Tribello , M. Bonomi , D. Branduardi , C. Camilloni , G. Bussi , Comput. Phys. Commun. 2014, 185, 604.

[73] C. R. Harris , K. J. Millman , S. J. van der Walt , R. Gommers , P. Virtanen , D. Cournapeau , E. Wieser , J. Taylor , S. Berg , N. J. Smith , R. Kern , M. Picus , S. Hoyer , M. H. van Kerkwijk , M. Brett , A. Haldane , J. F. Del Rio , M. Wiebe , P. Peterson , P. Gerard‐Marchant , K. Sheppard , T. Reddy , W. Weckesser , H. Abbasi , C. Gohlke , T. E. Oliphant , Nature 2020, 585, 357. 32939066 10.1038/s41586-020-2649-2 PMC7759461

[74] P. Virtanen , R. Gommers , T. E. Oliphant , M. Haberland , T. Reddy , D. Cournapeau , E. Burovski , P. Peterson , W. Weckesser , J. Bright , S. J. van der Walt , M. Brett , J. Wilson , K. J. Millman , N. Mayorov , A. R. J. Nelson , E. Jones , R. Kern , E. Larson , C. J. Carey , I. Polat , Y. Feng , E. W. Moore , J. VanderPlas , D. Laxalde , J. Perktold , R. Cimrman , I. Henriksen , E. A. Quintero , C. R. Harris , et al., Nat. Methods 2020, 17, 261. 32015543 10.1038/s41592-019-0686-2 PMC7056644

[75] N. Michaud‐Agrawal , E. J. Denning , T. B. Woolf , O. Beckstein , J. Comput. Chem. 2011, 32, 2319. 21500218 10.1002/jcc.21787 PMC3144279

[76] R. J. Gowers , M. Linke , J. Barnoud , T. J. E. Reddy , M. N. Melo , S. L. Seyler , J. Domanski , D. L. Dotson , S. Buchoux , I. M. Kenney , O. Beckstein , presented at Conference: Proc. of the 15th Python in Science Conf (SCIPY 2016); 2016‐07‐11 –2016‐07‐11; United States, 2019.

[77] W. Humphrey , A. Dalke , K. Schulten , J. Mol. Graph. 1996, 14, 33. 8744570 10.1016/0263-7855(96)00018-5

