# Supplementary material for: Rotation of the c‐Ring Promotes the Curvature Sorting of Monomeric ATP Synthases
Source: Adv Sci (Weinh). 2023 Sep 13;10(31):2301606. doi: 10.1002/advs.202301606 (PMC10625105; doi:10.1002/advs.202301606)
Supplement: Supplementary file 1 — Supporting Information [file ADVS-10-2301606-s007.pdf]

ADVANCED SCIENCE
Open Access

Supporting Information

for *Adv. Sci.*, DOI 10.1002/advs.202301606

Rotation of the *c*-Ring Promotes the Curvature Sorting of Monomeric ATP Synthases

David Valdivieso González, Marcin Makowski, M. Pilar Lillo, Francisco J. Cao-García, Manuel N. Melo, Víctor G. Almendro-Vedia and Iván López-Montero**

SUPPORTING INFORMATION

Rotation of the *c*-ring promotes the curvature sorting of monomeric ATP synthases

David Valdivieso González[1,2,†], Marcin Makowski[3,4†], M. Pilar Lillo[5], Francisco J. Cao-García[6,7], Manuel N. Melo[4], Víctor G. Almendro-Vedia[1,2,*] and Iván López-Montero[1,2,8*]

1. Departamento Química Física, Universidad Complutense de Madrid, Avda. Complutense s/n 28040 Madrid, Spain.

2. Instituto de Investigación Hospital Doce de Octubre (imas12), Avenida de Córdoba s/n, 28041 Madrid, Spain

3. Instituto de Medicina Molecular, Facultade de Medicina, Universidade de Lisboa, 1649-028 Lisbon, Portugal

4. Instituto de Tecnologia Química e Biológica António Xavier, Universidade Nova de Lisboa, Av. da República, 2780-157 Oeiras, Portugal.

5. Departamento Química Física Biológica, Instituto de Química-Física "Blas Cabrera" (CSIC), Serrano 119, 28006 Madrid, Spain.

6. Departamento de Estructura de la Materia, Física Térmica y Electrónica, Universidad Complutense de Madrid, Plaza de Ciencias, 1, 28040 Madrid, Spain

7. Instituto Madrileño de Estudios Avanzados en Nanociencia, IMDEA Nanociencia, C/ Faraday, 9, 28049 Madrid, Spain.

8. Instituto Pluridisciplinar. Paseo Juan XXIII, 1, 28040 Madrid, Spain.

[†] Both authors contributed equally
* corresponding author: ivanlopez@quim.ucm.es

Keywords: F_1F_o ATP synthase, *E.coli*, Giant vesicles, micromanipulation, lipid nanotubes.

Supplementary Results and Methods

DCCD inhibitory effect on F_1F_o ATP synthase. *N*,*N*-Dicyclohexylcarbodiimide (DCCD) is widely used as the gold standard inhibitor of F_1F_o-ATP synthase. The covalent binding to carboxyl residues of the c-ring blocks its rotation and allows for the uncoupling of the proton pumping and ATP hydrolysis/synthesis reactions. At high (mM) concentrations DCCD can also inhibit F_1 by reacting with the glutamic acid residues of the β subunits (βGlu192). However, this reaction is sensitive to the polar environment of the carboxyl groups (less favored in aqueous media) and requires a Mg^{2+}-free solution to bind F_1. Note that Mg^{2+} is present in external buffers used in sorting experiments. To fully discard the inhibition effect on F_1 in our experiments, we characterized independently the ATP hydrolysis activity of ATP synthase (F_1 activity) and the proton translocation activity (F_o rotation activity) in ATP synthase proteoliposomes.

Briefly, 166 μL of purified ATP synthase (0.2 mg/mL) were incubated with 200 μL of POPC (20 mM) for 45 min on ice and rapidly diluted to a volume of 2.5 mL with 5 mM HEPES (pH 7.4). Proteoliposomes carrying the ATP synthase were then collected by ultracentrifugation (MLA 80 rotor; 30 min at 298,906 × g) and resuspended in 60 μL of external buffer (15 mM HEPES pH 7.4, 5 mM $MgCl_2$, 55 mM NaCl, 100 mM glucose). For the F_o activity assessment, 6.8 μL of proteoliposomes were diluted with 143.2 μL of external buffer and 1.5 μL of ATP (100 mM in 1M HEPES, 100 mM $MgCl_2$, 100 mM K_2HPO_4, pH 7.5); and the proton translocation (rotation) was monitored by the change in the fluorescence emission intensity of pyranine, which was encapsulated at 150 μM into proteoliposomes (**Figure S1A**). The acidification of the lumen of proteoliposomes was deduced from the decrease of the fluorescence intensity of the probe emitted at $\lambda_{em} = 520\,nm$ upon excitation at $\lambda_{ex} = 468\,nm$ (Spectrofluorimeter Amingo AB2). Interestingly, the presence of DCCD (1 mM) prevented the luminal acidification of proteoliposomes, as visualized by the unaltered fluorescence emission intensity of pyranine over time. This observation suggests the inhibition of F_o, where the proton translocation across the membrane was prevented. The same proteoliposomes were tested for F_1 activity by the malachite green phosphate assay (**Figure S1B**), which detects the inorganic phosphate released from ATP during ATP hydrolysis [[1]]. Here, 35 μL of diluted proteoliposomes (see above) were resuspended in 105 μL of reaction buffer (60 mM HEPES pH 8; 6 mM $MgCl_2$, 60 mM KCl, 6μg/mL BSA and 1.2 mM DTT) and incubated for 1 hour at 37 ºC in the presence of 1 mM of ATP and different concentrations of DCCD (0, 0.1, 1 and 20 mM). The ATP hydrolysis activity was then assessed through malachite green assay. Compared to the control in the absence of DCCD, we observed an inhibited ATP hydrolysis of ATP synthase when incubated with a high dose of DCCD (20 mM, 50 % inhibition) whereas the presence of the inhibitor at lower concentrations (up to 1 mM) only prevented around 10% of ATP hydrolysis. Overall, the incubation of 1 mM DCCD in presence of Mg^{2+} maintains the F_1 ATP hydrolysis activity whereas the H^+ pumping activity of F_o is inhibited.

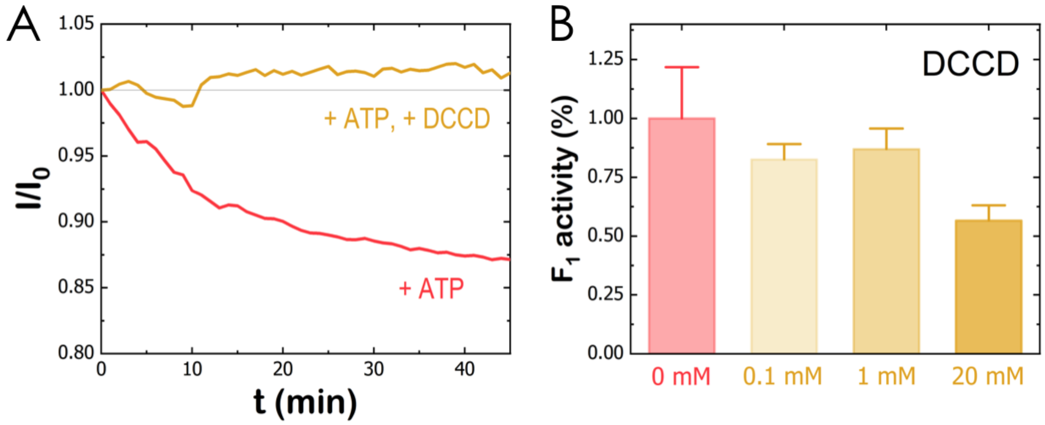

Figure S1. DCCD inhibition of ATP synthase reconstituted in proteoliposomes. A) F_o activity as measured by pyranine fluorescence assay. In the presence of external ATP, the inwards proton pumping activity is monitored by a decrease of the emission fluorescence intensity of enclosed pyranine in proteoliposomes whereas it remained unaltered in the presence of DCCD (1mM). **B)** ATP hydrolysis F_1 activity as measured by the green malachite assay. ATP synthase proteoliposomes are incubated with DCCD different concentrations. Note that the presence of Mg^{2+} (5 mM) partially prevents the inhibition of F_1 activity by DCCD up to 20 mM concentration.

Curvature sorting of Bacteriorhodopsin.

We further evaluated the proton transmembrane translocation as the origin for curvature sorting with control experiments using GUVs containing Bacteriorhodopsin (BR) from *Halobacterium salinarum*. BR is a bacterial light-driven proton pump that captures light energy to move protons across the membrane. BR is a trimer composed of seven transmembrane alpha helices. As a result, the cross-section of the transmembrane protein is a circular ring of $\approx 4\,nm$ in diameter, very similar to the dimensions of the c-ring of *E.coli* ATP synthase. The sorting ratio for BR was also compatible with a uniform distribution of the protein within the tube and the GUV, $S_{BR}^{dark} = 1.1 \pm 0.2\ (N = 7)$ (**Figure S2**). In addition, the curvature sorting of BR was assessed after illumination (see Material and Methods for experimental details) to induce a proton gradient across the GUV membrane (acid inside) (see **Figure S6** below). Again, the measured sorting parameter suggested a similar protein ratio in both the tube and the GUV, $S_{BR}^{ligth} = 1.1 \pm 0.2\ (N = 7)$. As for the ATP synthase (see **Figure 1D**), the absence of curvature sorting for passive and active BR might rely on the quite cylindrical profile of the protein. The tilt angle of BR at the protein –lipid interface is $\theta_{BR} \leq 1°$. As the protein radius is $R_{BR} \approx 1.8\,nm$, the spontaneous curvature is estimated to $c_{BR} \approx 0.009\,nm^{-1}$. For a small and rather symmetric protein like BR ($A_{BR} \approx 10\,nm^2$; $c_{BR} \approx 0.009\,nm^{-1}$) in a POPC membrane ($\kappa_l \approx 10k_BT$) [[2]] no measureable sorting would occur for physical curvatures ($c < 0.1\,nm^{-1}$). Note that, both the c-ring of ATP synthase and BR are able to crystalize within planar membranes [[3],[4]].

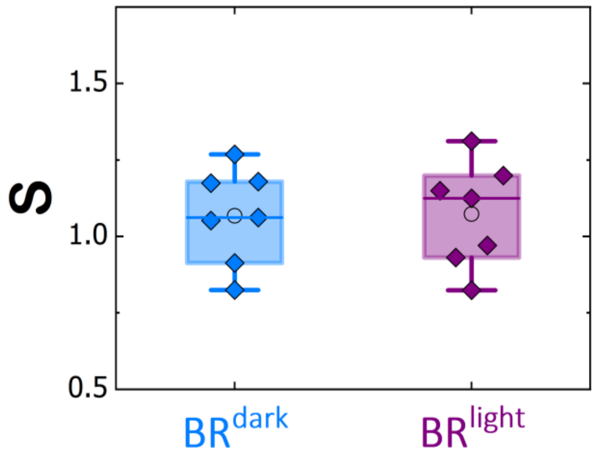

Figure S2. Box plot of the sorting ratio for tubes pulled from GUVs ($r_t = 50 \pm 20\,nm$) containing Bacteriorhodopsin (BR) from *Halobacterium salinarum* in the presence (light) and absence (dark) of illumination. The median is represented with a line; the box represents the 25th to 75th percentiles; and error bars show the 5th–95th percentile. The average sorting ratios (circles) are $S_{BR}^{light} = 1.1\ \pm 0.2\ (N = 7)$ and $S_{BR}^{dark} = 1.1\ \pm 0.1\ (N = 7)$.

Simulation torque and restraints

Reference points for the application of torque and distance restraints were always the centres of mass of groups of backbone particles. Torque involved defining four such backbone particle centres of mass per F_o group: i) of the entire *c*-ring; ii) of the 10 *c*-ring Pro43 (which lie at the top of the rotor); iii) of one of the 10 *c*-ring subunits; and iv) of the *a* and *b* subunits. Points i) and ii) defined the rotation axis; points i) and iii) defined one of the reference vectors for angle calculation, and points i) and iv) defined the second reference vector. The angle was computed using PLUMED [[5]] as the torsion, or dihedral angle, between the reference vectors along the rotation axis, and is therefore a measurement with period 2π. The PLUMED framework is able to treat this dihedral angle as a collective variable dependent on the positions of the backbone particles involved in the definition of points i) through iv). Any force imposed on this collective variable is then translated into biasing forces acting at the individual atom level resulting, in this case, in torque on the *c*-ring.

For preventing *c*-ring separation from the *a*/*b* subunits, four centre-of-mass reference points per F_o group were again employed: the entire *c*-ring, as for the torque, and low, mid, and top sections of the transversal helices of the *a* subunit. 600 kJ mol^{-1} nm^{-2} harmonic distance restraints were applied on each of the three distances between *c*-ring centre and *a* subunit segments.

For ease of reproducibility, the starting structures, topologies, and GROMACS and PLUMED setup files are also all included as Supporting Archive 2.

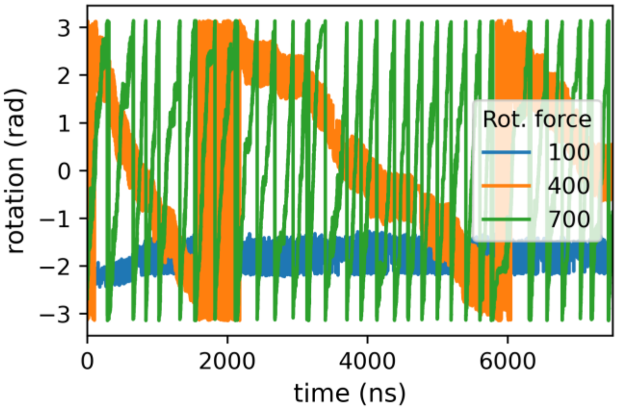

Figure S3. Rotation angle between the *c*-ring and the *a*/*b* subunits under different torques, as a function of simulation time. Torques were applied as constant forces of 100, 400, or 700 kJ mol^{-1} rad^{-1} (the rotations at 100 and 400 kJ mol^{-1} rad^{-1} are shown for the synthesis direction whereas the one at 700 kJ mol^{-1} rad^{-1} is shown for the hydrolysis direction). At the lowest torque, no rotation could be observed, whereas at the highest, the system enters a different, unencumbered, rotation mode. Also note the freedom afforded by the lower torques, which allows the *c*-ring to wiggle back and forth at each rotation step, yielding broadened angle distributions. No such freedom is allowed at the highest torque.

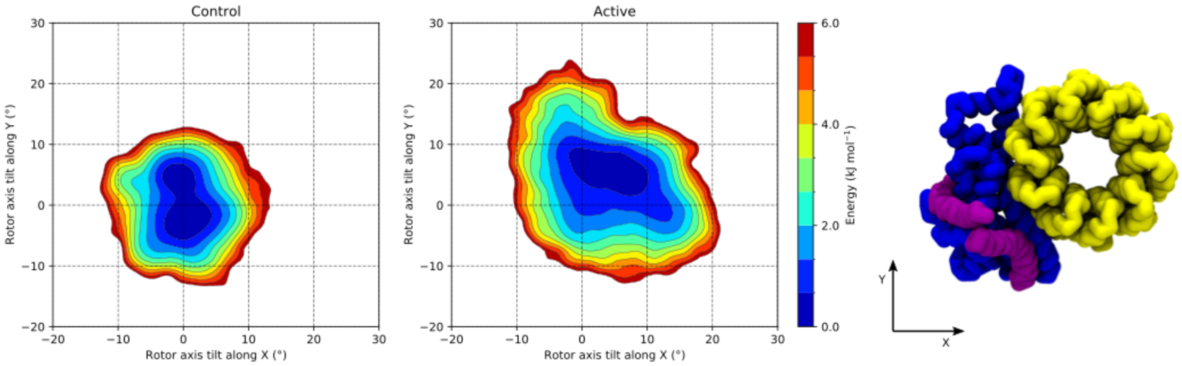

Figure S4. Distributions of *c*-ring axis tilt for control (passive) and active systems, depicted as energy surfaces — where lower energies indicate higher probabilities/frequencies. The surfaces are centred so that the average rotor orientation of the control systems lies at (0°, 0°). The depicted F_o top view indicates the placement of the *a*/*b* subunits (blue, purple) relative to the *c*-ring (yellow) in the chosen *X*-*Y* reference frame. When actively rotating, the *c*-ring rotor samples a much wider range of tilt angles than the passive control, with average position tilted between 5° and 10° from the control's average.

Protein–protein proximity preference

We observed a tendency for actively rotating ATP synthases to cluster spatially (measured in **Figure S5**–left within a 2 nm cut-off). Conversely, non-rotating synthases displayed a lower

propensity for overall spatial clustering, but when contacts did occur, they tended to be at closer distances (measured in **Figure S5–right** within a 0.7 nm cut-off). These observations show that such spatial affinity is likely mediated by the interplay with the membrane.

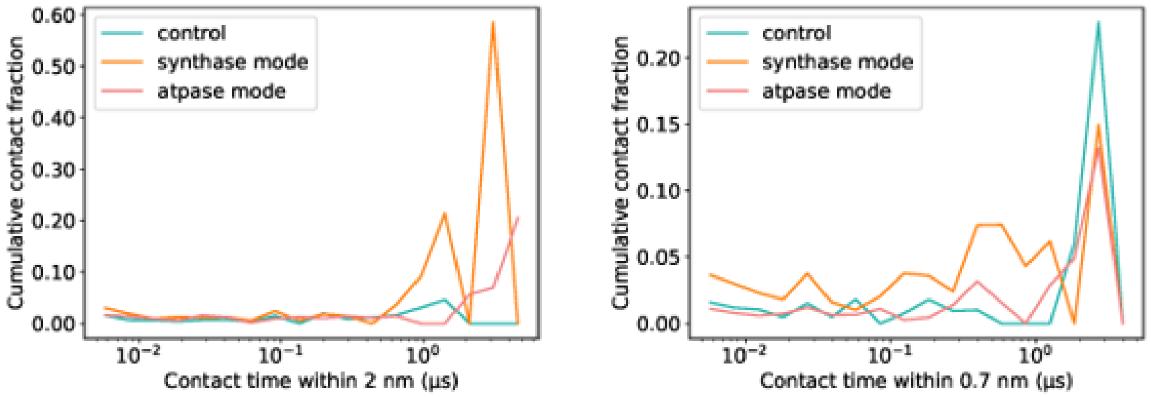

Figure S5. F_o–F_o contact time distributions at large (2 nm, left plot) or short (0.7 nm, right plot) contact cut-off distances. These plots represent histograms of logarithmically-sized contact duration bins, on a logarithmic *X*-scale. The *Y*-axis represents neither a contact count nor a contact frequency, but rather a sum of contact times within each of the histogram's bins, over all replicates, normalized for the total simulation time. This way, the histograms reflect the time fraction the systems spent having each contact duration. It is clear that having active rotation promotes F_o–F_o proximity, but also encourages shorter-lived close F_o–F_o contacts (below the 10^0 μs scale).

Pyranine assays for testing protein activity after reconstitution. An independent evaluation of a functional reconstitution is an essential step for the experimental validation of the sorting parameters obtained from tube pulling experiments. In our experimental setup, protein sorting was monitored for both ATP synthase and BR under different conditions. As both proteins are proton-pumping proteins, their activity was assessed using the pH–sensitive probe pyranine as a reporter to monitor the luminal acidification of proteo-GUVs (see experimental details in Material and Methods).

First, a strong acidification is observed after the external addition of ATP to ATP synthase proteo-GUVs (**Figure S6A**). The green luminal fluorescence decreased until it reached a plateau after 60 minutes of incubation. At 30 minutes, the decrease of fluorescence intensity corresponded to an approximate ΔpH range of 0.5 - 0.8 units as measured from the calibration curves previously measured by us [6]. In contrast, we did not observe any pyranine fluorescence intensity variation in ATP synthase GUVs in the absence of ATP (**Figure S6A**), suggesting a negligible spontaneous translocation of protons across the membrane. Interestingly, when experiments were performed in the presence of FCCP (20 μM) and ATP (1 mM), the fluorescence intensity of luminal pyranine remained unchanged, suggesting the FCCP-mediated dissipation of the proton gradient promoted by the active translocation of proton by the ATP

synthase (**Figure S6B**). An independent test for the FCCP-mediated proton translocation was assessed using proteo-GUVs in the presence of a pH gradient and the absence of ATP. Here, the fluorescence emission intensity of pyranine inside proteo-GUVs decreased rapidly overtime (**Figure S6B**). The pH gradient across the membrane provided by the external (pH 6.8) and internal media (pH 7.4) induced the inward proton translocation by FCCP. Again, we could estimate an approximate ΔpH change of 0.4 - 0.7 units from the average decrease of fluorescence intensity. Finally, the transmembrane proton pumping activity of BR was demonstrated in BR proteo-GUVs in the absence and presence of illumination. Upon illumination, BR-containing GUVs induced a time-dependent decrease of the luminal fluorescence intensity related to an internal acidification (**Figure S6C**). The decrease in fluoresce intensity corresponded to a pH gradient of about 0.3 - 0.8 units (acidic inside). Again, the passive permeability of protons across the membrane was negligible in BR proteo-GUVs without illumination (**Figure S6C**).

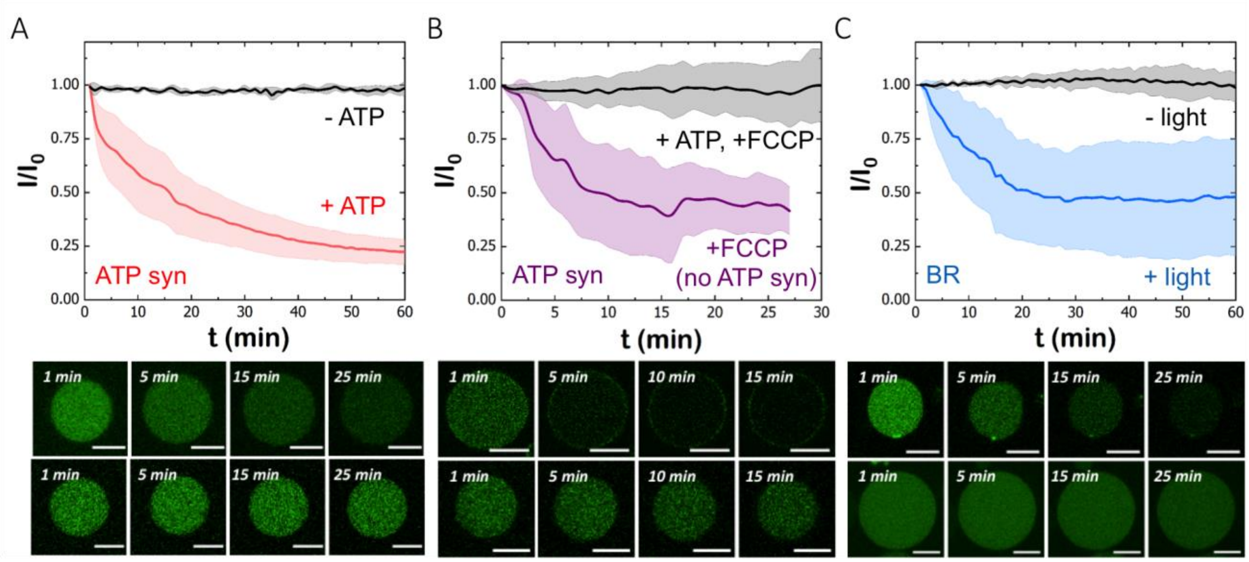

Figure S6. Proton translocation across GUVs by ATP synthase and BR as monitored by the luminal acidification of vesicles using the emission fluorescence intensity of pyranine. **A)** In the presence of external ATP (1 mM final concentration), the inwards proton pumping activity is monitored by a decrease of the emission fluorescence intensity of enclosed pyranine in ATP synthase GUVs ($N = 9$) whereas it remained unaltered in the absence of rotating conditions ($N = 3$). **Bottom:** representative images of ATP synthase proteoGUVs in the presence (up) and absence (down) of ATP. **B)** In the presence of external ATP (1 mM final concentration) and FCCP (20 μM), the proton accumulation in the lumen of ATP synthase proteo-GUVs is dissipated by the protonophore and the emission fluorescence intensity of enclosed pyranine remains constant overtime ($N = 5$). In contrast, FCCP is able to produce a proton gradient across of POPC GUVs in the presence of a pH gradient (basic inside) and the emission fluorescence intensity of enclosed pyranine decreases overtime ($N = 5$). **Bottom:** representative images of a POPC GUV in the presence of FCCP and a pH gradient (basic inside) (top) and an ATP synthase proteoGUV in the presence of ATP and FCCP (down). **C)** Time-evolution of the emission fluorescence intensity of pyranine enclosed in BR proteo-GUVs under

illuminating ($N = 10$) and non-illuminating ($N = 3$) conditions. **Bottom:** representative images of BR proteoGUVs in the presence (up) and absence (down) of illumination. Scale bars are 10 μm.

Calculation of the local curvature in CG simulations

The modelled systems with bilayers periodic in XY were built with a high X to Y length ratio. This caused curvature and undulations to emerge essentially along the X dimension, and we simplify our analysis by assessing it thus. The membrane patch was equally divided into 31 YZ slices; coordinates of the lipid tail carbons from each monolayer were binned into each X-slice and their Z positions averaged. At each slice, a final average between monolayer Z-averages was performed (separate averaging per monolayer prevents distortion in the final average due to lipid number asymmetry). Each slice was assigned a XZ coordinate pair, with X the midpoint of the slice in X and Z the final Z-average. As a result, a segmented chain with 31 XZ coordinate points was obtained for every frame (**Figure S7A, top**). The coordinates of the chain were used to circumscribe circles within three consecutive points to obtain the local curvature as the Menger curvature [7] — the reciprocal of the radius of the circumscribed circle (**Figure S7B**). This step was done iteratively for every point of the chain, also taking periodicity in X into consideration. Finally, the curvature within the bins occupied by a protein and within those devoid of protein (regions in blue and in green in **Figure S7A**, respectively) was obtained for all frames (**Figure S7A, bottom**). Averaged curvatures were obtained and confidence interval estimated by a subsampled bootstrap approach to correct for time-series autocorrelation.

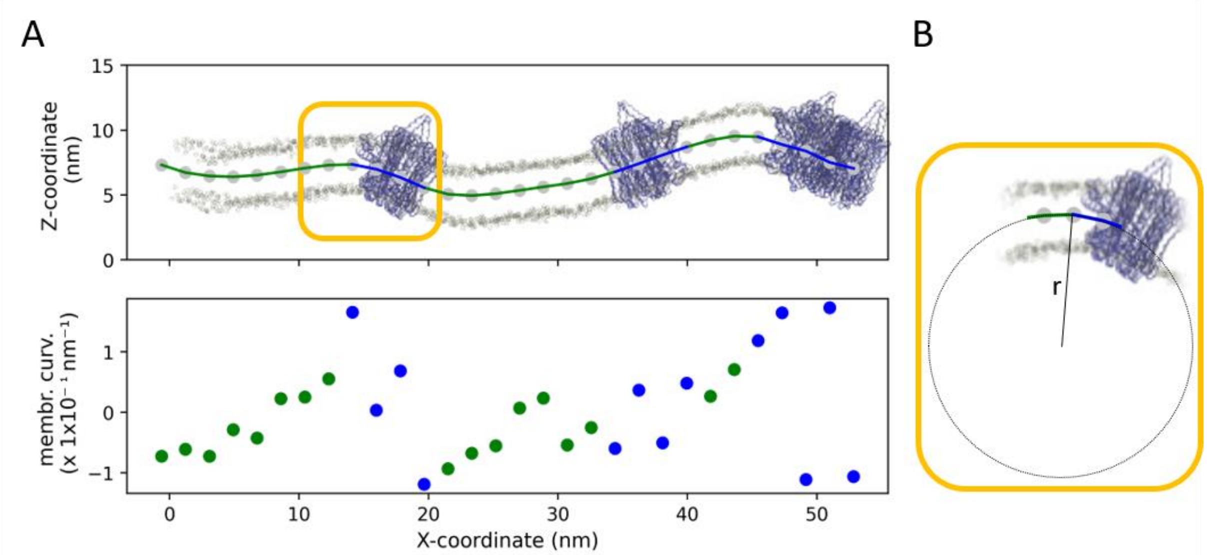

Figure S7. Illustration of the method used for membrane curvature calculation in the bilayer system. **A)** Illustration of binned Z-averaging along slices/bins in the X-axis; the lower graph indicates the Menger curvatures for each bin. **B)** Illustration of the Menger curvature (1/r), obtained using three consecutive points (bins) along the X-axis of the bilayer.

Videos. Coarse-grained molecular dynamics simulations of F_o domains in a POPC bilayer under rotating conditions; hydrolysis (**Video S1**) and synthesis (**Video S2**) and passive conditions (**Video S3**)

Description: Rotating F_o domains (yellow) induce considerable fluctuations on a POPC bilayer.

REFERENCES

[1] Lanzetta PA, Alvarez LJ, Reinach PS, Candia OA. An improved assay for nanomole amounts of inorganic phosphate. *Anal Biochem* **100,** 95 (1979)

[2] Manneville JB, Bassereau P, Lévy D, Prost J. Activity of Transmembrane Proteins Induces Magnification of Shape Fluctuations of Lipid Membranes. *Physical Review Letters* **82**, 4356-4359 (1999)

[3] Yamashita H, Voitchovsky K, Uchihashi T, Contera SA, Ryan JF, Ando T. Dynamics of bacteriorhodopsin 2D crystal observed by high-speed atomic force microscopy. *J Struct Biol* **167**, 153-158 (2009).

[4] Pogoryelov D, *et al.* Engineering rotor ring stoichiometries in the ATP synthase. *Proc Natl Acad Sci USA* **109**, 1599-1608 (2012).

[5] Tribello GA, Bonomi M, Branduardi D, Camilloni C, Bussi G. PLUMED 2: New feathers for an old bird. *Computer Physics Communications* **185**, 604 - 613 (2014).

[6] Almendro-Vedia VG, Natale P, Mell M, Bonneau S, Monroy F, Joubert F, Lopez-Montero I. Nonequilibrium fluctuations of lipid membranes by the rotating motor protein F1F0-ATP synthase. *Proc Natl Acad Sci USA* **114**, 11291- 11296 (2017).

[7] Ratliff HN. Cartesian Formulas for Curvature, Circumradius, and Circumcenter for Any Three Two-Dimensional Points. 2019.
